# China stroke surveillance report 2021

**DOI:** 10.1186/s40779-023-00463-x

**Published:** 2023-07-19

**Authors:** Wen-Jun Tu, Long-De Wang, on behalf of the Special Writing Group of China Stroke Surveillance Report, Feng Yan, Bin Peng, Yang Hua, Ming Liu, Xun-Ming Ji, Lin Ma, Chun-Lei Shan, Yi-Long Wang, Jing-Sheng Zeng, Hui-Sheng Chen, Dong-Sheng Fan, Yu-Xiang Gu, Guo-Jun Tan, Bo Hu, De-Zhi Kang, Jian-Min Liu, Yuan-Li Liu, Min Lou, Ben-Yan Luo, Su-Yue Pan, Li-Hua Wang, Jian Wu

**Affiliations:** 1 Department of Neurosurgery, Beijing Tiantan Hospital, Capital Medical University, Beijing, 100070 China; 2 School of Public Health, Peking University, Beijing, 100191 China

**Keywords:** Stroke, Epidemiological characteristics, Treatment, Prognosis, China

## Abstract

Since 2015, stroke has become the leading cause of death and disability in China, posing a significant threat to the health of its citizens as a major chronic non-communicable disease. According to the China Stroke High-risk Population Screening and Intervention Program, an estimated 17.8 million [95% confidence interval (CI) 17.6–18.0 million] adults in China had experienced a stroke in 2020, with 3.4 million (95% CI 3.3–3.5 million) experiencing their first-ever stroke and another 2.3 million (95% CI 2.2–2.4 million) dying as a result. Additionally, approximately 12.5% (95% CI 12.4–12.5%) of stroke survivors were left disabled, as defined by a modified Rankin Scale score greater than 1, equating to 2.2 million (95% CI 2.1–2.2 million) stroke-related disabilities in 2020. As the population ages and the prevalence of risk factors like diabetes, hypertension, and hyperlipidemia continues to rise and remains poorly controlled, the burden of stroke in China is also increasing. A large national epidemiological survey initiated by the China Hypertension League in 2017 showed that the prevalence of hypertension was 24.7%; the awareness, treatment, and control rates in hypertensive patients were: 60.1%, 42.5%, and 25.4%, respectively. A nationally representative sample of the Chinese mainland population showed that the weighted prevalence of total diabetes diagnosed by the American Diabetes Association criteria was 12.8%, suggesting there are 120 million adults with diabetes in China, and the awareness, treatment, and control rates in diabetic patients were: 43.3%, 49.0%, and 49.4%, respectively. The “Sixth National Health Service Statistical Survey Report in 2018” showed that the proportion of the obese population in China was 37.4%, an increase of 7.2 points from 2013. Data from 1599 hospitals in the Hospital Quality Monitoring System and Bigdata Observatory Platform for Stroke of China (BOSC) showed that a total of 3,418,432 stroke cases [mean age ± standard error (SE) was (65.700 ± 0.006) years, and 59.1% were male] were admitted during 2020. Of those, over 80% (81.9%) were ischemic stroke (IS), 14.9% were intracerebral hemorrhage (ICH) strokes, and 3.1% were subarachnoid hemorrhage (SAH) strokes. The mean ± SE of hospitalization expenditures was Chinese Yuan (CNY) (16,975.6 ± 16.3), ranging from (13,310.1 ± 12.8) in IS to (81,369.8 ± 260.7) in SAH, and out-of-pocket expenses were (5788.9 ± 8.6), ranging from (4449.0 ± 6.6) in IS to (30,778.2 ± 156.8) in SAH. It was estimated that the medical cost of hospitalization for stroke in 2020 was CNY 58.0 billion, of which the patient pays approximately CNY 19.8 billion. In-hospital death/discharge against medical advice rate was 9.2% (95% CI 9.2–9.2%), ranging from 6.4% (95% CI 6.4–6.5%) for IS to 21.8% for ICH (95% CI 21.8–21.9%). From 2019 to 2020, the information about 188,648 patients with acute IS receiving intravenous thrombolytic therapy (IVT), 49,845 patients receiving mechanical thrombectomy (MT), and 14,087 patients receiving bridging (IVT + MT) were collected through BOSC. The incidence of intracranial hemorrhage during treatment was 3.2% (95% CI 3.2–3.3%), 7.7% (95% CI 7.5–8.0%), and 12.9% (95% CI 12.3–13.4%), respectively. And in-hospital death/discharge against medical advice rate was 8.9% (95% CI 8.8–9.0%), 16.5% (95% CI 16.2–16.9%), and 16.8% (95% CI 16.2–17.4%), respectively. A prospective nationwide hospital-based study was conducted at 231 stroke base hospitals (Level III) from 31 provinces in China through BOSC from January 2019 to December 2020 and 136,282 stroke patients were included and finished 12-month follow-up. Of those, over 86.9% were IS, 10.8% were ICH strokes, and 2.3% were SAH strokes. The disability rate [% (95% CI)] in survivors of stroke at 3-month and 12-month was 14.8% (95% CI 14.6–15.0%) and 14.0% (95% CI 13.8–14.2%), respectively. The mortality rate [% (95% CI)] of stroke at 3-month and 12-month was 4.2% (95% CI 4.1–4.3%) and 8.5% (95% CI 8.4–8.6%), respectively. The recurrence rate [% (95% CI)] of stroke at 3-month and 12-month was 3.6% (95% CI 3.5–3.7%) and 5.6% (95% CI 5.4–5.7%), respectively. The Healthy China 2030 Stroke Action Plan was launched as part of this review, and the above data provide valuable guidelines for future stroke prevention and treatment efforts in China.

**Supplementary Information:**

The online version contains supplementary material available at 10.1186/s40779-023-00463-x.

## Background

Since 2015, stroke has become the leading cause of death and disability in China, posing a significant threat to the health of its citizens as a major chronic non-communicable disease [1]. The aging of the population and acceleration of urbanization have led to the popularity of unhealthy lifestyles, resulting in the widespread exposure of risk factors for cardiovascular and cerebrovascular diseases. As a result, the burden of stroke in China has experienced an alarming growth trend. However, stroke is a preventable and controllable disease, and early screening and active intervention can significantly improve patients’ outcomes. In 2009, the Stroke Screening and Prevention Project was launched by the former Ministry of Health, followed by the establishment of the China Stroke Prevention Project Committee (CSPPC) in 2011 [2]. The same year saw the launch of the National Major Public Health Project, “Screening and Intervention Project for High-risk Stroke Populations” [2]. The CSPPC proposed a 32-word prevention and control strategy, with a core emphasis on prevention and moving barriers forward [2, 3].

Since 2015, the CSPPC has been compiling the “China Stroke Surveillance Report” annually, which summarizes the public health, scientific research, clinical, screening, and intervention projects related to stroke prevention and control in China. This report serves as an essential platform for the release of authoritative stroke-related data in China, and provides the public with a window to understand the progress of stroke prevention and treatment in the country. The “China Stroke Surveillance Report 2021” refers to China’s latest major research results. More than 50 experts in stroke prevention and treatment contributed to the writing of the “China Stroke Surveillance Report 2021”, which was then discussed and revised by nearly 100 experts. The report primarily focuses on several areas, including the progress made in stroke diagnosis and treatment, the development of China’s stroke prevention and control system, the construction of the Bigdata Observatory Platform for Stroke of China (BOSC), data analysis of screening and intervention projects for high-risk stroke groups, prospects for stroke prevention and treatment, and the primary tasks and goals for future stroke prevention and control efforts.

## Methods and data sources

Stroke disease burden, epidemiological characteristics, and risk factors were extracted from published literature and the BOSC. Construction of stroke prevention and control system in China and China Stroke High-risk Population Screening and Intervention Program were extracted from BOSC. Findings of inpatient characteristics and treatment were extracted from BOSC and Hospital Quality Monitoring System (HQMS). Acute treatment of ischemic stroke (IS) patients and Hospital based Stroke Registry Study were also extracted from BOSC.

## Overview of stroke in China

As the largest developing country, China’s population accounts for one-fifth of the world’s total population, and its number of stroke patients ranks first in the world. The Global Burden of Disease (GBD) Study 2019 showed that there were 12.20 million incident cases of stroke, 101.00 million prevalent cases of stroke, and 6.55 million deaths from stroke in the world [4], and the data in China were 3.94 million, 28.76 million and 2.19 million [5]. Furthermore, the “China Stroke Report 2019” released by the National Center for Medical Quality Control of Neurological Diseases in 2020, pointed out that stroke has become the leading cause of premature death and disease burden [6]. GBD data also show that the disability-adjusted life years (DALYs) caused by stroke are higher than most other diseases, such as heart disease, respiratory disease, and digestive system disease [7].

## Stroke disease burden

In the past 30 years, with the development of the social economy, the lifestyle has undergone tremendous changes, and the overall incidence of stroke in China has shown a rising trend. With the accelerated aging of the population, the increasing prevalence of unhealthy lifestyles among residents, and the widespread exposure to stroke risk factors, the burden of stroke disease in China is on an explosive growth trend. It is estimated that the incidence of cerebrovascular disease events in China in 2030 will increase by about 50% compared with 2010 [8].

### Incidence

GBD data showed that from 2010 to 2019, the incidence of IS increased from 129/100,000 to 145/100,000, while the incidence of hemorrhagic stroke dropped from 61/100,000 to 45/100,000 [9]. In a cross-sectional study of 676,394 participants aged 40 years and older, the overall age-standardized-prevalence, incidence, and mortality rate of stroke in Chinese mainland were estimated to be 2.61%, 505.2/100,000, and 343.4/100,000, respectively, in the year 2020 [10].

### Prevalence

GBD data show that the overall prevalence of IS in China is rising. From 2010 to 2019, the prevalence of IS increased from 1100/100,000 to 1256/100,000, while the prevalence of hemorrhagic stroke decreased from 232/100,000 to 215/100,000 [9]. The data from “China Stroke High-risk Population Screening and Intervention Program 2020” showed that among the 676,394 community residents over 40 years old, there were 22,976 stroke patients, with a standardized prevalence rate of 2610/100 000, and the prevalence of IS was 2270/100,000, and the prevalence of hemorrhagic stroke was 390/100,000 [10].

### Recurrence rate

A population-based cohort study included adults aged 35–74 years without disability recruited to the China Kadoorie Biobank (CKB), and the results showed that 41% had a recurrent stroke at 5 years and recurrence at a year was 17% [11]. The CSPPC investigated 12-month stroke fatality, disability, and recurrence rates after the first-ever stroke through a prospective nationwide hospital-based cohort study {36,250 first-ever stroke patients from 194 hospitals were recruited [median age was 65 (IQR 56–73) years and 61.4% were male]}. The 12-month stroke recurrence rate was 5.7% [95% confidence interval (CI) 5.5–6.0%] for stroke survivors, ranging from 2.5% (95% CI 1.7–3.3%) for subarachnoid hemorrhage (SAH) to 6.4% (95% CI 6.0–6.7%) for IS [12].

### Mortality rate

The “China Health Statistical Yearbook 2020” showed that in 2019, the crude stroke death rate for rural residents was 158.63/100,000, and for urban residents was 129.41/100,000 [13]. Stroke has become the second-largest cause of death among rural residents in China (accounting for 22.94% of all causes of death) and the third cause of death for urban residents (accounting for 20.61% of all causes of death). From 2010 to 2019, the crude stroke mortality rate of urban residents did not change significantly, while the crude mortality rate of rural residents showed a fluctuating upward trend and was consistently higher than that of urban residents during the same period [13]. The CKB data showed that 28-day mortality was 3% for IS, 47% for intracerebral hemorrhage (ICH), 19% for SAH, and 24% for unspecified stroke [11]. The CSPPC investigated 12-month stroke fatality, disability, and recurrence rates after the first-ever stroke through a prospective nationwide hospital-based cohort study and the data showed that the in-hospital death rate was 1.9% (95% CI 1.7–2.0%) for stroke inpatients, ranging from 0.9% (95% CI 0.8–1.1%) for IS to 5.1% (95% CI 4.6–5.6%) for ICH. The 12-month fatality rate was 8.6% (95% CI 8.3–8.9%) for discharged stroke patients, ranging from 6.0% (95% CI 5.7–6.3%) for IS to 17.7% (95% CI 16.7–18.7%) for ICH [12].

### DALYs

GBD data showed that the DALYs of IS in China had a slight downward trend as a whole from 2010 (1209/100,000) to 2019 (1148/100,000), and the DALYs of hemorrhagic stroke also showed a significant downward trend, from 1671/100,000 in 2010 to 1142/100,000 in 2019 [14]. In 2019, there were 45.9 million (39.8–52.3 million) DALYs due to stroke in China [5].

### Economic burden of stroke

The “China Health Statistical Yearbook 2020” showed that from 2010 to 2019, the number of discharged patients, especially IS, and per capita hospitalization costs were all on the rise. In 2019, there were 4,335,072 patients with IS and 611,709 patients with hemorrhagic stroke discharge, an increase of 4 times and 2 times, respectively, compared with 2010 [13]. This growth trend reflects that the development of population aging structure and the prevalence of risk factors and stroke continues to rise; Alternatively, this may indicate that stroke-related popular science education has become more widespread, leading to increased public awareness of stroke and higher rates of doctor visits. As a result, in 2019, the per capita hospitalization cost of IS and hemorrhagic stroke patients were Chinese Yuan (CNY) 9809 and CNY 20,106, an increase of 37% and 82%, respectively, compared with 2010 [13]. Therefore, we estimated that the medical cost of hospitalization for stroke in 2019 was CNY 54.8 billion, of which the patient paid approximately CNY 18.3 billion (33.4%).

## Epidemiological characteristics of stroke

The epidemiological characteristics of stroke in China are mainly as follows: 1) age characteristics, younger-onset (average age 65 years); 2) regional differences, high in the North and low in the South, and prominent in the middle; 3) differences between urban and rural areas, high in rural areas; 4) gender differences, high in men.

### Age characteristics

The incidence of stroke in China shows a trend in younger people. In China, the average age of stroke patients is about 65 years old, which is lower than about 75 years old in developed countries [7]. GBD data showed that the proportion of patients with IS and hemorrhagic stroke under 70 in China remained basically unchanged from 2010 to 2019, at about 57% [14]. The data from “Screening and Intervention Project for High-risk Stroke Populations 2012–2016” showed that the average age of stroke patients aged 40 years and older at the first onset of stroke was from (60.9 ± 0.04) to (63.4 ± 0.05) years old, and 40–64 years old patients accounted for more than 66.6% of the first onset age group (Internal data not yet published).

### Geographical distribution

The overall distribution of stroke incidence, prevalence, and mortality in China is “high in the North, low in the South, and prominent in the middle”. According to the research results released by the China Health and Nutrition Survey in 2020: from 1997 to 2015, the incidence of stroke in Northern China was the highest (417/100,000 person-years), followed by the central region (287/100,000 person-years), the lowest in the South (195/100,000 person-years) [15]. Data released by national epidemiological survey of stroke in China showed that from 2012 to 2013, the incidence of stroke (365/100,000 person-years) and mortality (159/100,000 person-years) in Northeast China were the highest, followed by the central region (incidence rate of 326/100,000 person-years, the mortality rate was 154/100,000 person-years), the Southern region is the lowest (the incidence rate was 155/100,000 person-years, the mortality rate was 65/100,000 person-years); the stroke prevalence rate was the highest in the central region (1550/100,000), followed by the Northeast region (1450/100,000), and the Southern region with the lowest (625/100,000) [16]. In fact, a stroke belt of high stroke incidence existing in 9 provincial areas of North and West China had been suggested [16].

### Urban and rural differences

According to the National Health Service Survey, the prevalence of stroke in rural areas of China was lower than that in urban areas from 1993 to 2008, but the prevalence in rural areas has increased rapidly in the past 10 years. In 2013, it was the same as that in urban areas, and it surpassed the urban prevalence rate in 2018 [17]. According to the “China Health Statistical Yearbook 2020”, from 2010 to 2019, the stroke mortality rate in rural areas continued to exceed that in urban areas, and the gap between the two increased significantly [13]. The increasing burden of a stroke may be related to the high prevalence of hypertension, diabetes, and hyperlipidemia with suboptimal control [18].

### Gender differences

The burden of stroke in men is higher than that of women in China. “China Stroke Prevention and Control Report 2020” showed that from 2017 to 2020, the prevalence of stroke in men was higher than that of women in China, and the prevalence in men showed an increasing trend year by year, while the trend in women was relatively stable [19]. The “China Health Statistical Yearbook 2020” results showed that the crude stroke mortality rate for both urban and rural residents from 2010 to 2019 was higher for men than for women [13]. The stroke disease burden is higher in men than that in women, which may be related to the prevalence of risk factors such as smoking and alcohol consumption among men [20].

## Stroke risk factors

Stroke risk factors are divided into non-interventional risk factors and modifiable risk factors. Non-interventional risk factors include age, race, and genetic factors. Modifiable risk factors are risk factors for major interventions in stroke prevention, including hypertension, diabetes, dyslipidemia, heart disease, smoking, alcohol intake, diet, overweight or obesity, insufficient activity, and psychological factors. Data from the “Screening and Intervention Project for High-risk Stroke Populations” showed that hypertension, diabetes, obesity, unreasonable diet, and insufficient physical activity are closely related to the risk of stroke [21]. The INTERSTROKE study showed that 10 potentially modifiable risk factors are collectively associated with about 90% of the population attributable risks (PAR) of stroke in each major region [22]. SIREN study also suggested that 98.2% (95% CI 97.2–99.0%) of adjusted PAR of stroke was associated with 11 potentially modifiable risk factors [23].

### Hypertension

Hypertension is the most important modifiable risk factor for stroke [24]. A large national epidemiological survey (May Measurement Month Project) initiated by the China Hypertension League in 2017 showed that among the 364,000 Chinese residents who participated in the survey, the prevalence of hypertension was 24.7%; the awareness, treatment, and control rates in hypertensive patients (*n* = 89,925) were 60.1%, 42.5%, and 25.4%, respectively; in hypertensive patients receiving antihypertensive therapy (*n* = 38,207), the control rate was 59.8% [21]. It is worth noting that there are significant differences in the epidemiological distribution of hypertension in different regions of China, with the highest prevalence in Northeast and North China, which is consistent with the distribution of stroke prevalence from North to South [25]. Although the prevalence of hypertension in rural and urban areas of China is similar, the awareness, treatment, and control rates of hypertension patients in rural areas are significantly lower than those in urban areas [26]. In the community-based Kailuan Study, 16,006 participants aged 18 to 40 years and examined at baseline in 2006/2007 underwent 2-yearly follow-up examinations from 2016 to 2017, and the data showed that the newly defined category of stage 1 hypertension in young untreated Chinese adults aged < 40 years at baseline was associated with an increased risk for cardiovascular disease, stroke, and all-cause mortality [27]. Other studies have found that uncontrolled hypertension after the onset of IS or transient ischemic attack could increase the 1-year poor functional outcome and the risk of stroke recurrence [28, 29].

### Diabetes

In 2019, the International Diabetes Federation updated the Global Diabetes Report (http://www.diabetesatlas.org/), and the report showed that the number of adults (20–79 years old) with diabetes in China is 116.4 million (35.5 million patients are over 65 years old), suggesting that China with the largest number of diabetes patients in the world. A nationally representative sample of the Chinese mainland population from 2015 to 2017, including 75,880 participants aged 18 and older showed that the weighted prevalence of total diabetes diagnosed by the American Diabetes Association criteria was 12.8% (95% CI 12.0–13.6%) among adults living in China, suggesting there were 120 million adults with diabetes in China and the awareness, treatment, and control rates in diabetic patients were 43.3%, 49.0%, and 49.4%, respectively [30]. The Chinese Chronic Disease and Risk Factor Monitoring Study conducted in 2013 showed that the prevalence of diabetes in Chinese residents aged 18 and above was about 10.9%, and the awareness, treatment, and control rates in diabetic patients were 36.5%, 32.2%, and 49.2%, respectively [31]. In addition, awareness and treatment rates were higher among older, female, and urban populations, whereas control rates were higher among younger and urban people [31]. The “Sixth National Health Service Statistical Survey Report in 2018” results showed that the prevalence of self-reported diabetes in the population over 15 years old in China in 2018 was 5.3%, an increase of 1.8 points compared with 2013 [32]. About 14% of adult stroke patients in China had diabetes mellitus [10]. The 7-year follow-up results of the CKB found that compared with the non-diabetic population, the all-cause mortality [relative risk (*RR*) = 2.00, 95% CI 1.93–2.08] and stroke-related mortality (*RR* = 1.98, 95% CI 1.81–2.17) were increased in diabetic population [33].

### Dyslipidemia

Dyslipidemia is one of the critical risk factors for atherosclerosis, and it is also an independent risk factor for stroke [34]. The prevalence of dyslipidemia among residents aged 18 and over in China is about 34%, while the awareness rate (31.0%), treatment rate (19.5%), and control rate (8.9%) of dyslipidemia patients are all under low level [35]. The results of the Monitoring Study on Chronic Diseases and Risk Factors in China showed that among the high-risk groups of atherosclerotic cardiovascular and cerebrovascular diseases, 74.5% of the participants did not reach the low-density lipoprotein cholesterol (LDL-C) target, and only 5.5% of them received lipid-lowering drugs treatment; in the very high-risk population, 93.2% of participants were not at LDL-C targets, of whom only 14.5% received lipid-lowering medication [36]. A prospective study involving 6 cohorts of 267,500 Chinese residents with a median of 2,295,881 person-years of follow-up found that each 1 mmol/L increase in total blood cholesterol [hazard ratio (*HR*) = 1.08, 95% CI 1.05–1.11], low-density lipoprotein cholesterol (*HR* = 1.08, 95% CI 1.04–1.11) and triglyceride concentration (*HR* = 1.07, 95% CI 1.05–1.09) can significantly increase the risk of IS [37]. This study also found an increased risk of hemorrhagic stroke is associated with abnormal total cholesterol and high-density lipoprotein cholesterol, but not low-density lipoprotein cholesterol and triglyceride levels [37]. Recent studies also suggested that elevated plasma lipoprotein-associated phospholipase A2 levels were associated with an increased risk of stroke [38], and with early neurological deterioration after IS [39].

### Atrial fibrillation

Atrial fibrillation increases the risk of stroke [40]. China Stroke High-risk Population Screening and Intervention Program 2014 showed that the overall standardized prevalence of atrial fibrillation in China’s community population over 40 years old was 2.31% [41]. The China Registry of Atrial Fibrillation (CRAF) study from July to December 2012 consecutively enrolled 4161 patients with atrial fibrillation from 111 hospitals, showing that there were 3562 (85.6%) patients were non-valvular atrial fibrillation, 599 (14.4%) patients were rheumatic valvular atrial fibrillation, and 76.5% of patients with non-valvular atrial fibrillation and 72.8% of patients with valvular atrial fibrillation were at high risk of stroke (CHA2DS2-VASc ≥ 2) [42]. CRAF also showed that the overall anticoagulation rate in atrial fibrillation was 37.1% (0.9% of novel anticoagulants), and the anticoagulation use rate of patients with non-valvular atrial fibrillation was significantly lower than that of patients with valvular atrial fibrillation (25.6% vs. 57.3%, *P* < 0.001) [42]. A community-based survey of 47,841 adults (age ≥ 45 years) in 7 geographic regions of China between 2014 and 2016 showed that the prevalence of atrial fibrillation among residents over the age of 45 was 1.8%. The awareness rate of atrial fibrillation patients aged 45 to 54 was 65.3%, the awareness rate of atrial fibrillation patients over 75 years old was 53.9%, varied between sex (men 58.5%, women 68.8%) and residency status (urban 78.3%, rural 35.3%). In addition, only 6.0% of patients with high-risk atrial fibrillation received anticoagulation [43].

### Smoking

Smoking is one of the risk factors for stroke and poor prognosis [44, 45]. In addition, the GBD Study 2017 estimated 380 million smokers in China [46]. “China Smoking Harmful Health Report 2020” showed that there were more than 300 million smokers in China in 2018, the smoking rate of people over 15 years old in China was 26.6%, and the smoking rate of males was 50.5%; more than 1 million people lose their lives to tobacco every year, and if no effective action is taken, it is expected to increase to 2 million per year by 2030 and 3 million per year by 2050 [47]. Smoking increases the risk of cardiovascular disease and related deaths [47], and smoking cessation reduces stroke incidence and improves prognosis and recurrence [48, 49]. Epstein et al. [50] found that smoking cessation within 6 months of stroke or transient ischemic attack could reduce the risk of a composite of stroke recurrence, myocardial infarction, and death within 5 years (*HR* = 0.66, 95% CI 0.48–0.90). The association between family history and the risk of IS was more pronounced among smokers but did not differ between those who had quit smoking for more than 10 years or those who had never smoked [51]. In addition, a study had suggested that passive smoking may be associated with stroke and increased disease burden (attributed DALYs 45,789 person-years) [52].

### Alcohol intake

Heavy alcohol intake is associated with an increased risk of total stroke [53]. More than one-third of men and 2% of women in China have the habit of drinking alcohol every week [54]. According to the survey results of the “Sixth National Health Service Statistical Survey Report in 2018”, the drinking rate of the population over the age of 15 in the survey areas in China was 27.6% in 2018 [32], and higher consumption of alcohol and tobacco in Northern than Southern China [55]. Heavy drinking (> 294 g/week in men and > 196 g/week in women) was associated with an increased risk of stroke [Odds Ratio (*OR*) = 2.09, 95% CI 1.64–2.67] compared with non-drinkers or ex-drinkers [56]. A study based on 22,691 rural residents in China found that high-dose alcohol consumption (> 721 g/week) increased the risk of stroke [57]. Another survey based on 23,433 male community residents found that even low-dose alcohol consumption (average daily alcohol intake < 15 g) significantly increased the risk of IS [58]. Heavy drinking can also lead to increased stroke-related mortality, and a 15-year follow-up study exploring the relationship between drinking behavior and mortality in Chinese men showed that compared with non-drinkers, long-term drinkers had a 16% increase in stroke-related mortality (*HR* = 1.16, 95% CI 1.08–1.24) and the risk of death increases with increasing alcohol consumption [59].

### Unreasonable diet

The impact of nutrition at the food group and dietary pattern level on stroke had been proposed, and adherence to the Mediterranean and prudent nutritional patterns reduced the risk of stroke, whereas the Western dietary pattern was associated with increased stroke risk [60]. With the rapid development of the social economy, the dietary structure and eating habits of Chinese residents have undergone tremendous changes (the dietary characteristics have gradually tended to be high in calories, fat, and sugar), and this dietary change has led to an increased risk of chronic diseases such as cardiovascular disease and cerebrovascular disease [61]. A traditional Northern diet (mainly refined grains and salted vegetables) is associated with an increased risk of stroke compared with a standard Southern Chinese diet (mainly rice, vegetables, and fruits) [44]. Excessive long-term sodium intake can cause high blood pressure, cardiovascular disease, and stroke [62]. The Kailuan study divided participants into low (salt < 6 g/d), moderate (salt 6–10 g/d, equivalent sodium 2.4–4 g/d), and high intake (salt > 10 g/d) based on the salt intake of residents in 2006, the results showed that the risk of IS was reduced in the low and moderate salt intake group (*HR* = 0.76, 95% CI 0.63–0.92), but there was no significant change in the risk of hemorrhagic stroke (*HR* = 0.84, 95% CI 0.55–1.29) [63]. The Salt Substitute and Stroke Study—a 5-year cluster randomized controlled trial, demonstrated that replacing regular salt with a reduced-sodium added-potassium salt substitute reduced the risks of stroke, major adverse cardiovascular events, and premature death among individuals with prior stroke or uncontrolled high blood pressure living in rural China [64] and replacing regular salt with salt substitute also was a cost-saving intervention for the prevention of stroke and improvement of quality of life [65]. In addition, a modeling study published in the *British Medical Journal* in 2020 also pointed out that in China, the use of high-potassium and low-sodium salts instead of ordinary sodium salts can reduce blood pressure and prevent 365,000 stroke occurrences and 208,000 stroke-related deaths [66].

### Overweight or obesity

Overweight and obesity can increase the risk of stroke by co-acting with common risk factors such as hypertension, diabetes, and dyslipidemia and promote stroke occurrence through other mechanisms such as changes in endothelial cell function, thrombosis, and systemic inflammation [67–70]. A study published in *The Lancet* in 2016 showed that in 2014, there were nearly 90 million obese people in China [71]. According to the survey results of the “Sixth National Health Service Statistical Survey Report in 2018”, the proportion of the obese [body mass index (BMI) ≥ 24 kg/m^2^] population in China was 37.4%, an increase of 7.2 points from 2013 [32]. A systematic review of 44 prospective cohort studies with 4,432,475 participants showed a J-shaped dose–response relationship between BMI and stroke risk, with a 1.10-fold increase in stroke risk for every 5-unit increase in BMI (*RR* = 1.10, 95% CI 1.06–1.13), and the risk did not increase significantly in the range of BMI < 24 kg/m^2^ but increased dramatically with the increase of BMI in the range of BMI > 25 kg/m^2^ [72]. In addition, the results of a large epidemiological study based on 26,185 participants in 2020 pointed out that after 11.8 years of follow-up, a total of 1507 participants developed stroke, and the risk of stroke increased with BMI increased [73]. Zhejiang Metabolic Syndrome Cohort and Kailuan Study included 102,037 participants and finished a 10-year follow-up. The results showed that compared with patients with normal metabolism and normal weight, patients with abnormal metabolism and obesity had a 2.11-fold increased risk of stroke (*HR* = 2.11, 95% CI 1.50–2.97) [74].

### Physical inactivity

Physical inactivity is a risk factor for stroke, and regular physical activity reduces stroke risk [75, 76]. The results of the “Sixth National Health Service Statistical Survey Report in 2018” showed that in 2018, residents over 15 years old reported consciously participating in physical exercise per week was 49.9%, which was 22.1 percentage points higher than that in 2013 [32]. A questionnaire survey conducted in Southwest China suggested that insufficient physical activity was closely associated with a high prevalence of stroke [77]. To reduce the risk of cardiovascular and cerebrovascular diseases, the 2019 U.S. guidelines for primary prevention of cardiovascular disease recommend that no less than 150 min of moderate-intensity aerobic physical activity or no less than 75 min of high-intensity aerobic physical activity should be performed every week; and some moderate-to-high-intensity exercise can be beneficial, even if the above-mentioned intensities cannot be achieved [78]. Physical activity can prevent the occurrence of stroke and promote recovery and prevent recurrence in stroke patients [79, 80].

### Psychological factors

Adverse psychological factors increase the risk of stroke and stroke mortality [81, 82]. China Kadoorie Biobank Collaborative Group included 487,377 people aged 30–79 years without previous stroke, heart disease, or tumor and finished 7 years of follow-up; the data showed that after adjusting for gender, age, marital status, and other related factors, severe depression might increase the risk of stroke (*HR* = 1.15, 95% CI 0.99–1.33), and there was a dose–response relationship between the risk of stroke and the number of depressive symptoms, and compared with the group with the number of symptoms “0–2”, the number of symptoms “6” (*HR* = 1.33, 95% CI 1.01–1.74) and the number of symptoms “7” (*HR* = 1.47, 95% CI 1.04–2.08) were associated with significantly increased stroke risk [83]. In addition, a mendelian randomization study showed that major depression may be positively associated with the risk of small-artery occlusive stroke [84]. A prospective cohort study including 221,677 participants reported that psychological distress had a strong, dose-dependent, positive association with myocardial infarction and stroke in men and women [85].

### Other relevant factors

#### Hyperhomocysteinemia

Homocysteine (HCY), a thiol-containing amino acid, is an essential intermediate in methionine and cysteine metabolism [86]. Factors such as congenital enzyme deficiencies, chronic renal and hepatic dysfunction, and drug therapy can lead to elevated plasma levels of homocysteine, known as hyperhomocysteinemia (Hhcy) [87]. A systematic review and meta-analysis estimated that the prevalence of Hhcy (> 15 μmol/L) in the Chinese population was 27.5% [88]. A nationwide survey was conducted from October 2018 to September 2019, including 110,551 residents ≥ 40 years of age from 31 provinces in Chinese mainland, to assess the prevalence of Hhcy, and the results showed that a total of 28,633 participants (25.9%) were defined as Hhcy and the prevalence ranged from 7.9% in Shanghai to 56.8% in Tianjin [89]. It is closely associated with the risk of stroke and is one of the risk factors for poor prognosis [90, 91]. A recent systematic review of 11,061 subjects in 10 studies showed that Hhcy increases the risk of stroke [92]. Stroke risk may be reduced by vitamin supplementation, which is associated with homocysteine metabolism. A systematic review of 389,938 participants in 12 prospective studies showed that daily intake of folic acid by 100 μg (pooled *RR* = 0.94, 95% CI 0.90–0.98) or vitamin B6 by 0.5 mg (pooled *RR* = 0.94, 95% CI 0.89–0.99) can significantly reduce the risk of stroke [93]. The China Stroke Primary Prevention Study included 20,702 hypertensive patients without previous stroke or myocardial infarction, and the results suggested that compared with enalapril alone, enalapril combined with folic acid (0.8 mg/d) can significantly reduce the risk of stroke (*HR* = 0.79, 95% CI 0.68–0.93). After adjusting for age, sex, research center, and blood pressure, hypertensive patients with Hhcy were more likely to suffer from stroke than those without Hhcy, and the benefit was more pronounced in hypertensive patients with cysteinemia [94].

#### Environmental factors

Air pollution can increase the risk of stroke and stroke mortality [95–97]. Air pollution is divided into gaseous pollution and particulate pollution according to the form, and the representatives of gaseous pollutants are sulfur dioxide, nitrogen dioxide, carbon monoxide, and ozone [98]. The mixture of solid and liquid particles in the atmosphere from different sources, suspended particulate matter (PM), which cannot be distinguished by the naked eye, is called haze in meteorology. Particulate pollutants can be divided into PM_10_, PM_2.5_, PM_1_, and PM_0.1_ according to their aerodynamic equivalent diameters, among which PM_2.5_ is most closely related to health [99, 100]. GBD research showed that the atmospheric concentration of PM_2.5_ in China continued to increase from 1900 to 2013, and in 2013, China ranked second among 79 countries [101]. A study based on 5 hospitals found that residents with short-term exposure to PM_10_, PM_2.5_, and PM1 atmospheres had an increased risk of IS (*RR* = 1.005, 1.007, and 1.014, respectively) [102]. The China-PAR project that included 117,575 adult residents in 15 provinces (autonomous regions and municipalities) found that residents living in a high-concentration PM_2.5_ atmospheric environment for a long time had an increased risk of new stroke and the risk of stroke was increased by 13%, including a 20% increase in IS and a 12% increase in hemorrhagic stroke [103]. Qian et al. [104] found that the association between PM_2.5_ pollution and hemorrhagic stroke risk was more pronounced in diabetic patients.

In addition, a previous study also suggested that elevated ozone concentration may increase the risk of stroke in some specific populations (every 10 μg/m^3^ increase in exposure to ozone in the 2 to 3 d before the onset of the disease), the risk of stroke increased by 7.8% in rural residents, 6.5% in males and 5.8% in people over 60 years old, but there was no significant effect on the population as a whole [105].

## Construction of stroke prevention and control system in China

### Construction of regional stroke prevention and control network

According to the strategic requirements of the “Healthy China 2030” Plan, CSPPC implements the work strategy of “combining prevention and treatment”, promotes the integrated development of “prevention, treatment, management, and rehabilitation” for stroke as a whole, builds a stroke prevention and control work system consisting of the National Health Commission, CSPPC, provincial and municipal health administrative departments (including traditional Chinese medicine management departments), hospitals, primary medical and health institutions, disease control centers, emergency institutions, and other units, and explores the “four-in-one” full-process health management service model of population stroke screening and prevention, emergency first aid, standardized treatment, and rehabilitation follow-up.

### Stroke screening and prevention base hospital construction

The CSPPC has selected 327 regional leading tertiary hospitals in 31 provinces (autonomous regions and municipalities) as the base hospitals to carry out the project “China Stroke High-risk Population Screening and Intervention Program”. The selection of hospitals is mainly based on recruitment and voluntary participation. The CSPPC will confirm the application and award the license after evaluation. During the confirmation process, regional representation, which represents the national geographical distribution and social and economic status in China, will also be considered. Relying on 327 stroke prevention and treatment base hospitals, implement pre-hospital “Stroke High-risk Population Screening and Acute Stroke Patients First Aid”, in-hospital “High-risk Screening and Multidisciplinary Joint Diagnosis and Treatment”, and post-hospital “Follow-up Intervention”, which could cover the whole life cycle [3]. In 2020, more than 240 base hospitals from 31 provinces participated in a stroke screening and intervention program and completed a total of 1.256 million tasks, including 818,500 screening and intervention tasks for community populations and 443,500 intervention tasks, and the high-risk detection rate was 21.2% (95% CI 21.1–21.3%).

### Brain and heart health managers

From 2019 to 2020, CSPPC trained and certified 1370 brain and heart health managers. These personnel participated in the in-hospital screening project, especially the work of pre-hospital education and high-risk screening, in-hospital individualized intervention and health education, and out-of-hospital rehabilitation guidance and comprehensive management.

### Construction of Stroke Center in China

In 2011, the CSPPC proposed a work plan to construct a stroke-based hospital. In 2016, the General Office of CSPPC issued the Work Plan of Stroke Center, providing more precise directions to improve stroke treatment, control system development, and implement comprehensive stroke treatment and control strategies and measures [106–109]. The structure of the Chinese Stroke Center is composed of two levels and four layers. The two levels refer to the Advanced Stroke Center (hospital level: level III and above) and the Stroke Prevention Center (hospital level: level II and above). The advanced class is divided into two layers: the Demonstration Advanced Stroke Center and the Advanced Stroke Center. The Stroke Prevention Center is divided into the Demonstration Stroke Prevention Center and the Stroke Prevention Center [106]. In 2020, 635 stroke centers participated in reporting the treatment information of stroke patients to the BOSC, and 1,532,109 cases were submitted [109]. The distribution ratio of various stroke cases showed that IS accounted for 72.72%, cerebral hemorrhage accounted for 15.46%, SAH accounted for 5.00%, and others accounted for 6.85%. The number of intravenous thrombolysis cases (rt-PA and Urokinase) in the national advanced stroke center was 74,187 (60,442 with rt-PA and 13,187 with Urokinase), and the median time from admission to administration (Door to Needle Time, DNT) was 43 min (rt-PA: 42 min; Urokinase: 45 min). The median DNT time was 2 min shorter than last year when the number of intravenous thrombolysis cases increased significantly. In 2020, 47,409 cases of intravenous thrombolysis were carried out in the stroke prevention and treatment center, of which 13,255 cases were urokinase thrombolysis, accounting for 27.9%, which was higher than the 12.3% rate of urokinase intravenous thrombolysis in advanced stroke centers. In 2020, the development of interventional recanalization technology for acute IS showed an increasing trend compared to the previous year, with 31,131 cases completed. Carotid endarterectomy and carotid artery stenting are important assessment indicators for constructing advanced stroke centers. Driven by the construction of stroke centers, these two technologies have developed rapidly, and the number of surgeries has increased significantly. According to the data reported by BOSC, a total of 6788 carotid endarterectomy cases were carried out in 2020, and the serious complication rate was 1.50%, which remained at a low level (< 3%) as determined by international guidelines; a total of 21,796 cases of carotid artery stenting were carried out in 2020, and the incidence of serious complications is 2.30%, suggesting the safety of the operation is guaranteed.

### Construction of China’s Stroke First Aid Map

The Stroke First Aid Map is a process management model for the integrated configuration, specific link, and rapid delivery of medical resources. The stroke map (an app that allows patients to locate the nearest suitable stroke therapy hospital) and stroke Green Channel create a three 1-h gold rescue circle, abbreviated as “1-1-1” (onset to call time < 1 h, pre-hospital transfer time < 1 h, and DNT < 1 h) [10]. As of December 31, 2020, 154 cities, 1917 medical institutions, and emergency centers in 26 provinces have become “Stroke First Aid Map” units.

### Standardized training and promotion of stroke prevention and treatment

China’s stroke prevention and treatment work has achieved positive results. However, according to the national survey data on stroke diagnosis and treatment, China’s thrombolysis and thrombectomy rates still lag behind developed countries [110]. Unbalanced, non-standard, and heterogeneous development of technology is still prominent. In particular, the county-level stroke centers in China will only cover 32.5% of all counties by the end of 2020. In 2020, the Office of the CSPPC selected and determined the first batch of training bases for intravenous thrombolysis/arterial thrombectomy technology. The main task is to carry out thrombolysis/thrombectomy technology training for medical institutions across the country and jointly promote the standardized and homogeneous development of thrombolysis/thrombectomy technology.

## China Stroke High-risk Population Screening and Intervention Program

### Background of the project

China Stroke High-risk Population Screening and Intervention Program is an ongoing population-based screening project that enrolled around 0.8 million community-dwelling adults aged ≥ 40 years each year from all 31 provinces in Chinese mainland [2, 3]. The pilot project began in 2011 and covered 31 provinces in 2013. The sampling process consists of two steps. In the first stage of sampling, at each city, we randomly selected one community according to their size (at least 2000 residents aged ≥ 40 years) and population stability, in which potential participants were invited to the survey by local staff from stroke base hospitals. In the next stage, in each selected location, one village with a total population of at least 2000 residents 40 years or older was selected by using a random-sampling method.

### Project implementation

In 2020, the project covered 31 provinces, 229 base hospitals, and 459 screening and intervention sites. After data cleaning, there were 676,394 participants aged 40 years or older included in the survey [mean age ± standard deviation (SD): (59.7 ± 11.0) years; 395,122 (58.4%) females], covering 31 provinces, 219 base hospitals, and 396 screening intervention project sites [10].

### The detection rate of high-risk groups for stroke

About 172,043 high-risk groups were confirmed, with a standardized detection rate of 23.77% (men: 28.65%; women: 18.82%). The proportion of high-risk groups in the surveyed population continue to increase, and the work of stroke prevention and control still faces enormous pressure. It is necessary to actively strengthen stroke education and prevention and control. The data shows that the proportion of different risk factors in the newly screened high-risk population in 2020 is the same as that of the total population. The top three are hypertension, dyslipidemia, and lack of exercise, and the standardized detection rates are 79.18%, 70.53%, and 45.67%, respectively. Hypertension accounts for nearly 80% of the high-risk population, reflecting that the effect of high blood pressure prevention and control is not ideal, and high blood pressure prevention and control still have a long way to go.

### Stroke patient data analysis

Data were weighted to adjust for differential probabilities of selection and differential response and post-stratify the sample to adjust the population distribution. Post-stratification weights were adjusted for residence, geographic location, sex, and age using the 2010 China census data. The screening data in 2020 included a total of 676,394 people over the age of 40, including 22,974 stroke patients, with a standardized prevalence, incidence, and mortality of 2.61% (95% CI 2.57–2.64%) (2.94% in males and 2.27% in females; 2.69% in urban and 2.54% in rural), 505.2/100,000 person-years (95% CI 488.5–522.0) and 343.4/100,000 person-years (95% CI 329.6–357.2), respectively. Based on a previous literature report [16] and our findings, we estimated the disease burden of stroke in the Chinese adult population. Overall, the standardized prevalence, incidence, and stroke-related mortality in the Chinese adult population ≥ 18 years of age was 1.60% (95% CI 1.58–1.62%), 310.0/100,000 person-years (95% CI 299.8–320.3) and 210.7/100,000 person-years (95% CI 202.2–219.2), suggesting that 17.8 million (95% CI 17.6–18.0 million) of the Chinese adult population had a stroke, 3.4 million (95% CI 3.3–3.5 million) of the Chinese adult population had a first-ever stroke and another 2.3 million (95% CI 2.2–2.4 million) of the stroke-related death in 2020 [10]. In addition, 12.5% (95% CI 12.4 –12.5%) of stroke survivors were defined as disabled [modified Rankin Scale (mRS) > 1], representing 2.2 million (95% CI 2.1–2.2 million) stroke-related disabilities in 2020.

## Inpatient characteristics and treatment

Stroke patients (IS, ICH, SAH) were from hospitals where the HQMS and BOSC intersect. The HQMS, launched in 2011 by the National Health Commission of the People’s Republic of China, is an official data collection system, and the “Medical Record Home page” is collected [111]. The BOSC, previously referred to as the China Stroke Center Data-Sharing Platform, was established in 2020 by the CSPPC in collaboration with the National Population and Health Science Data Sharing Platform. Its primary purpose is to facilitate population-wide stroke risk factor screening, intervention management, and monitoring of the treatment status of hospitalized stroke patients [2, 3, 109]. In December 2021, a total of 1599 hospitals co-existed on both platforms.

In 2020, a total of 3,418,432 stroke cases [mean age ± standard error (SE) was (65.700 ± 0.006) years, and 59.1% were male] were identified from 1599 hospitals. Over 80% were IS (81.9%), 14.9% were ICH strokes, and 3.1% were SAH strokes (Table 1). The proportion of IS ranges from 33.9% in Tibet to 88.9% in Hainan, the proportion of ICH strokes increased from 9.5% in Hainan to 44.7% in Tibet, and the ratio of SAH strokes increased from 1.6% in Hainan to 21.4% in Tibet (Additional file 1: Fig. S1).

**Table 1:** Basic and clinical information of hospitalized stroke patients in China, 2020

Characteristics	Stroke (*n* = 3,418,432)	IS (*n* = 2,801,380)	ICH (*n* = 510,928)	SAH (*n* = 106,124)
Tertiary hospital [*n* (%)]	2,345,061 (68.6)	1,884,153 (67.3)	370,868 (72.6)	90,040 (84.8)
Age (years, mean ± SE)	65.700 ± 0.006	67.200 ± 0.007	62.000 ± 0.020	59.400 ± 0.040
Male [*n* (%)]	2,021,672 (59.1)	1,656,663 (59.1)	322,138 (63.0)	42,871 (40.4)
Married [*n* (%)]	3,027,221 (88.6)	2,486,113 (88.7)	447,219 (87.5)	93,889 (88.5)
Han [*n* (%)]	3,220,821 (94.2)	2,644,984 (94.4)	476,535 (93.2)	99,302 (93.6)
Length of stay (d, mean ± SE)	10.700 ± 0.005	10.500 ± 0.006	17.200 ± 0.030	14.800 ± 0.007
Hospitalization expenditures (CNY, mean ± SE)	16,975.6 ± 16.3	13,310.1 ± 12.8	34,708.3 ± 69.2	81,369.8 ± 260.7
Out-of-pocket expenses (CNY, mean ± SE)	5788.9 ± 8.6	4449.0 ± 6.6	11,570.8 ± 45.7	30,778.2 ± 156.8
Rehabilitation costs (CNY, mean ± SE)	155.6 ± 0.5	153.1 ± 0.6	417.9 ± 2.5	232.5 ± 4.6
Ventilator use [*n* (%)]	51,293 (1.5)	14,636 (0.5)	26,752 (5.2)	9905 (9.3)
Discharge outcome [*n* (%)]				
Medical discharge	2,966,934 (86.8)	2,522,000 (90.0)	369,032 (72.2)	75,902 (71.5)
Medical order transfer	39,858 (1.2)	25,021 (0.9)	10,754 (2.1)	4083 (3.8)
Transfer to grass-roots hospitals	52,621 (1.5)	43,764 (1.6)	7704 (1.5)	1153 (1.1)
Discharge against medical advice	266,608 (7.8)	160,404 (5.7)	88,111 (17.2)	18,093 (17.0)
Death	47,850 (1.4)	19,605 (0.7)	23,621 (4.6)	4624 (4.4)
Unclear	44,561 (1.3)	30,586 (1.1)	11,706 (2.3)	2269 (2.1)

*IS* ischemic stroke, *ICH* intracerebral hemorrhage, *SAH* subarachnoid hemorrhage, *CNY* Chinese Yuan

As shown in the Table 1, the mean ± SE of length of stay was (10.700 ± 0.005) d, ranging from (10.500 ± 0.006) in IS to (17.200 ± 0.030) in ICH and ventilator use rate was 1.5% (95% CI 1.5–1.5%), ranging from 0.5% (95% CI 0.5–0.5%) in IS to 9.3% (95% CI 9.2–9.5%) in SAH. The mean ± SE of hospitalization expenditures was CNY (16,975.6 ± 16.3), ranging from (13,310.1 ± 12.8) in IS to (81,369.8 ± 260.7) in SAH, and out-of-pocket expenses were CNY (5788.9 ± 8.6), ranging from (4449.0 ± 6.6) in IS to (30,778.2 ± 156.8) in SAH. We estimated that the medical cost of hospitalization for stroke in 2020 was 58.0 billion, of which the patient paid approximately 19.8 billion.

In-hospital death rate was 1.4% (95% CI 1.4–1.4%), ranging from 0.7% (95% CI 0.7–0.7%) in IS to 4.6% (95% CI 4.6–4.7%) in ICH and in-hospital death/discharge against medical advice rate was 9.2% (95% CI 9.2–9.2%), ranging from 6.4% (6.4–6.5%) for IS to 21.8% for ICH (21.8–21.9%) (Additional file 1: Fig. S2).

The mean ± SE of hospitalization expenditures for strokes increased from CNY (11,176.2 ± 47.3) in Gansu to (57,539.7 ± 108.5) in Tibet (Additional file 1: Fig. S3). As shown in Additional file 1: Fig. S4, the mean ± SE of out-of-pocket expenses for strokes increased from CNY (57.1 ± 21.5) in Tibet to (12,934.5 ± 184.7) in Beijing. The out-of-pocket expense rate for stroke in China was reported to be 42.3% (95% CI 41.6–43.1%). This rate varied across regions, with Tibet recording the lowest rate of 0.1% (95% CI 0.1–0.1%), and Fujian having the highest rate of 55.9% (95% CI 55.2–56.5%). As shown in Additional file 1: Fig. S5, the stroke out-of-pocket expense rate in 7 provinces (Shaanxi, Gansu, Xinjiang, Jilin, Ningxia, Qinghai, and Fujian) exceeded the national average level.

In-hospital death rate for IS was 0.7% (95% CI 0.7–0.7%), ranging from 0.2% (95% CI 0.2–0.2%) in Hunan and 0.2% (95% CI 0.2–0.3%) in Hainan to 2.4% (95% CI 2.2–2.6%) in Shanghai, and in-hospital death/discharge against medical advice rate was 6.4% (95% CI 6.4–6.5%), ranging from 1.5% (95% CI 1.5–1.5%) in Gansu to 13.8% (95% CI 13.5–14.1%) in Tianjin (Additional file 1: Fig. S6). In-hospital death rate for ICH was 4.6% (95% CI 4.6–4.7%), ranging from 1.2% (95% CI 1.0–1.4%) in Hunan to 14.1% (95% CI 12.5–15.6%) in Xinjiang Production and Construction Corps, and in-hospital death/discharge against medical advice rate was 21.8% (95% CI 21.8–21.9%), ranging from 11.2% (95% CI 10.9–11.6%) in Jiangsu to 34.9% (95% CI 34.4–35.4%) in Hebei (Additional file 1: Fig. S7). In-hospital death rate for SAH was 4.4% (95% CI 4.2–4.5%), ranging from 1.1% (95% CI 0.8–1.4%) in Hunan to 19.2% (95% CI 14.8–23.6%) in Xinjiang Production and Construction Corps, and in-hospital death/discharge against medical advice rate was 21.4% (95% CI 21.1–21.7%), ranging from 8.2% (95% CI 6.8–9.6%) in Beijing to 38.9% (95% CI 36.6–41.2%) in Chongqing (Additional file 1: Fig. S8).

## Acute treatments of IS patients

From 2019 to 2020, the information about 188,648 patients with acute IS [(mean age ± SD): (68.7 ± 11.4) years; male: 64.4%] receiving intravenous thrombolytic therapy (IVT), 49,845 patients [(mean age ± SD): (68.3 ± 11.4) years; male: 64.2%] receiving mechanical thrombectomy (MT), and 14,087 patients [(mean age ± SD): (68.7 ± 11.4) years; male: 64.2%] receiving bridging (IVT + MT) were collected through BOSC (Table 2).

**Table 2:** Basic and clinical information on different treatments for acute ischemic stroke

Characteristics	IVT (*n* = 188,648)	MT (*n* = 49,845)	Bridging (IVR + MT) (*n* = 14,087)
Sex-male [*n* (%)]	121,574 (64.4)	31,999 (64.2)	9049 (64.2)
Age [years, (mean ± SD)]	68.7 ± 11.4	68.3 ± 11.4	68.7 ± 11.4
Age [*n *(%)]			
≤ 40 years	844 (0.5)	297 (0.6)	80 (0.6)
40–60 years	43,148 (22.9)	11,825 (23.7)	3221 (22.9)
≥ 60 years	144,656 (76.7)	37,723 (75.7)	10,786 (76.6)
Race-Chinese Han [*n* (%)]	183,857 (97.5)	48,622 (97.6)	13,706 (97.3)
Hospital grade-tertiary [*n* (%)]	142,550 (75.6)	47,871 (96.1)	13,121 (93.2)
Seven Regions of China [*n* (%)]			
North China	36,397 (19.3)	4590 (9.2)	1226 (8.7)
Northeast	21,583 (11.4)	4536 (9.1)	1060 (7.5)
Central China	55,668 (29.5)	17,654 (35.4)	5081 (36.1)
East China	33,822 (17.9)	8023 (16.1)	2310 (16.4)
South China	13,751 (7.3)	7320 (14.7)	2139 (15.2)
Southwest	20,082 (10.7)	5083 (10.2)	1567 (11.1)
Northwest	7345 (3.9)	2639 (5.3)	704 (5)
Time from onset to hospital arrival within 2 h [*n* (%)]	135,543 (75.4)	21,049 (47.3)	10,656 (79.8)
Transported to hospital by EMS [*n* (%)]	69,603 (36.9)	24,846 (49.9)	7633 (54.2)
BMI (kg/m^2^, mean ± SD)	23.9 ± 24.4	23.9 ± 3.7	23.8 ± 3.5
Systolic pressure (mmHg, mean ± SD)	153.0 ± 23.1	148.9 ± 24.6	149.4 ± 24.2
Diastolic pressure (mmHg, mean ± SD)	87.5 ± 14.1	85.9 ± 15.1	86.0 ± 14.8
Toast classification in ischemic stroke			
1-LAA	93,478 (49.6)	31,062 (62.3)	8809 (62.5)
2-CE	23,104 (12.3)	14,244 (28.6)	4191 (29.8)
3-SVO	63,321 (33.6)	2163 (4.3)	518 (3.7)
4-SOE	1394 (0.7)	678 (1.4)	149 (1.1)
5-SUE	7337 (3.9)	1687 (3.4)	419 (3)
NIHSS score at admission [median (IQR)]	6 (3–12)	15 (10–20)	15 (11–22)
Acute phase treatment			
DNT (min, mean ± SD)	166.0 ± 1325.3	–	64.9 ± 849.8
ONT (min, mean ± SD)	248.6 ± 5933.7	–	171.5 ± 653.5
Thrombolytic complications [*n* (%)]	21,772 (11.5)	–	1251 (8.9)
Intracranial hemorrhage [*n* (%)]	6089 (3.2)	–	542 (3.8)
DPT (min, mean ± SD)	–	322.6 ± 6235.9	195.4 ± 1057.6
OPT (min, mean ± SD)	–	562.9 ± 6702.7	297.6 ± 796.8
Thrombectomy complications [*n* (%)]	–	6893 (13.8)	2152 (15.3)
Intracranial hemorrhage [*n* (%)]	–	3861 (7.7)	1273 (9.0)
Reasons for not thrombolysis—over time window [*n* (%)]	–	16,608 (33.3)	-
Length of stay (d, mean ± SD)	11.2 ± 18.4	14.6 ± 26.0	14.7 ± 16.1
Discharge outcome [*n *(%)]			
Medical discharge	159,101 (84.3)	36,585 (73.4)	10,382 (73.7)
Medical order transfer	6537 (3.5)	1843 (3.7)	488 (3.5)
Transfer to grass-roots hospitals	3407 (1.8)	1760 (3.5)	464 (3.3)
Discharge against medical advice	13,542 (7.2)	5733 (11.5)	1549 (11.0)
Death	3233 (1.7)	2511 (5.0)	821 (5.8)
Unclear	2828 (1.5)	1413 (2.8)	383 (2.7)

*IVT* intravenous thrombolytic therapy, *MT* mechanical thrombectomy, *SD* standard deviation, *IQR* interquartile range, *BMI* body mass index, *EMS* Emergency Medical Services, *LAA* large artery atherosclerosis, *SVO* small vessel occlusion, *CE* cardioembolism, *SOE* stroke of other determined etiology, *SUE* stroke of undetermined etiology, *NIHSS* NIH Stroke Scale score, *DNT* Door to Needle Time, *ONT* Onset to Needle Time, *DPT* door-puncture-time, *OPT* onset-puncture-time

Approximately 75.6% of patients receiving IVT came from tertiary hospitals, and there were 96.1% and 93.2% for MT and bridging. The NIH Stroke Scale score at admission for those three types of stroke patients were 6 (IQR 3–12), 15 (IQR 10–20), and 15 (IQR 11–22), respectively. The proportion of patients with large vessel occlusion was 49.6%, 62.3%, and 62.5%, respectively. The incidence of intracranial hemorrhage during treatment was 3.2% (95% CI 3.2–3.3%), 7.7% (95% CI 7.5–8.0%), and 12.9% (95% CI 12.3–13.4%), respectively. In-hospital death rate was 1.7% (95% CI 1.7–1.8%), 5.0% (95% CI 4.8–5.2%), and 5.8% (95% CI 5.4–6.2%), respectively. In-hospital death/discharge against medical advice rate was 8.9% (95% CI 8.8–9.0%), 16.5% (95% CI 16.2–16.9%), and 16.8% (95% CI 16.2–17.4%), respectively (Table 2 and Additional file 1: Fig. S9).

**Fig. 1:**
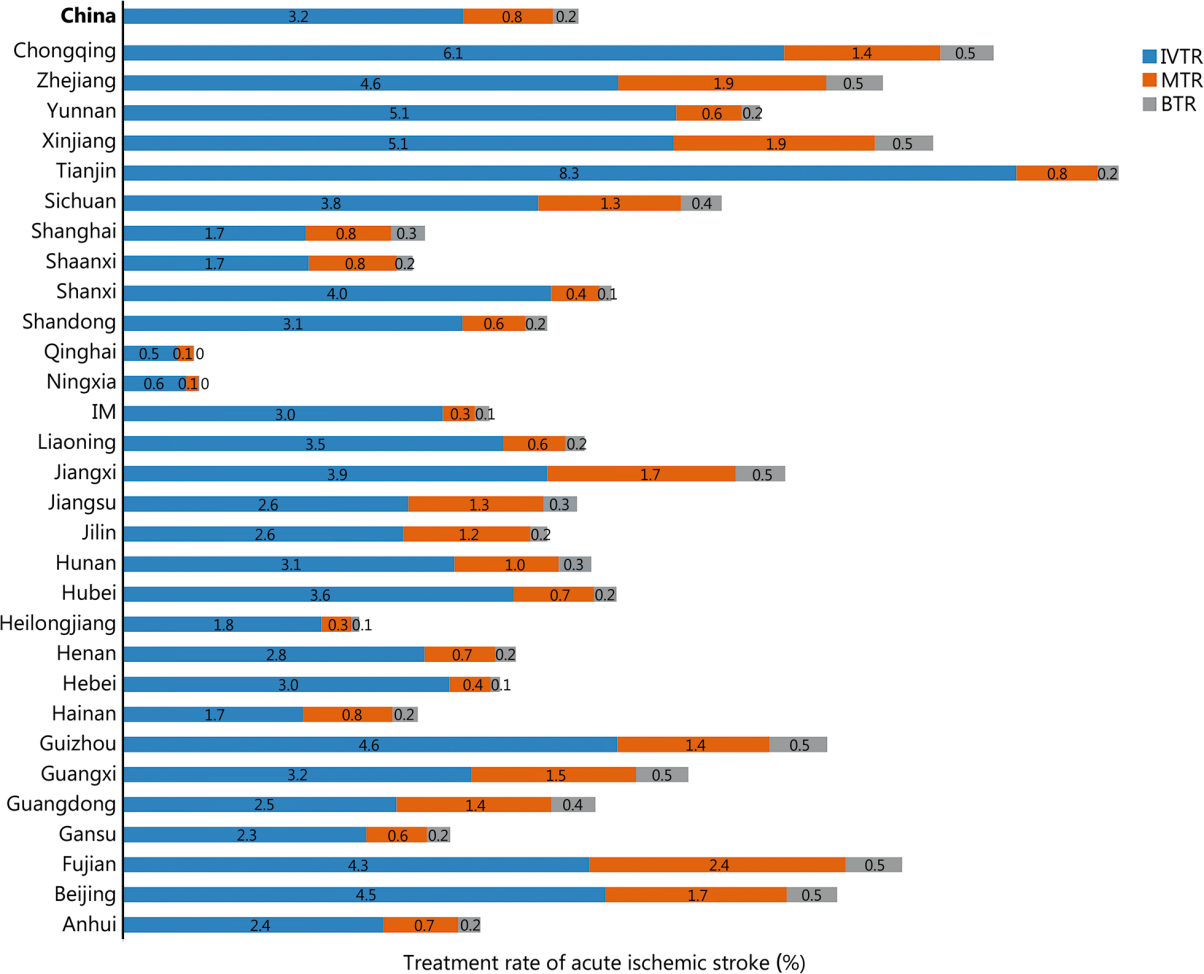
Rates of acute therapies in different provinces, 2019–2020 (%). Missing data from Xinjiang Production and Construction Corps and Tibet. IM Inner Mongolia, IVTR intravenous thrombolysis rate, MTR mechanical thrombectomy rate, BTR bridging therapy rate

**Fig. 2:**
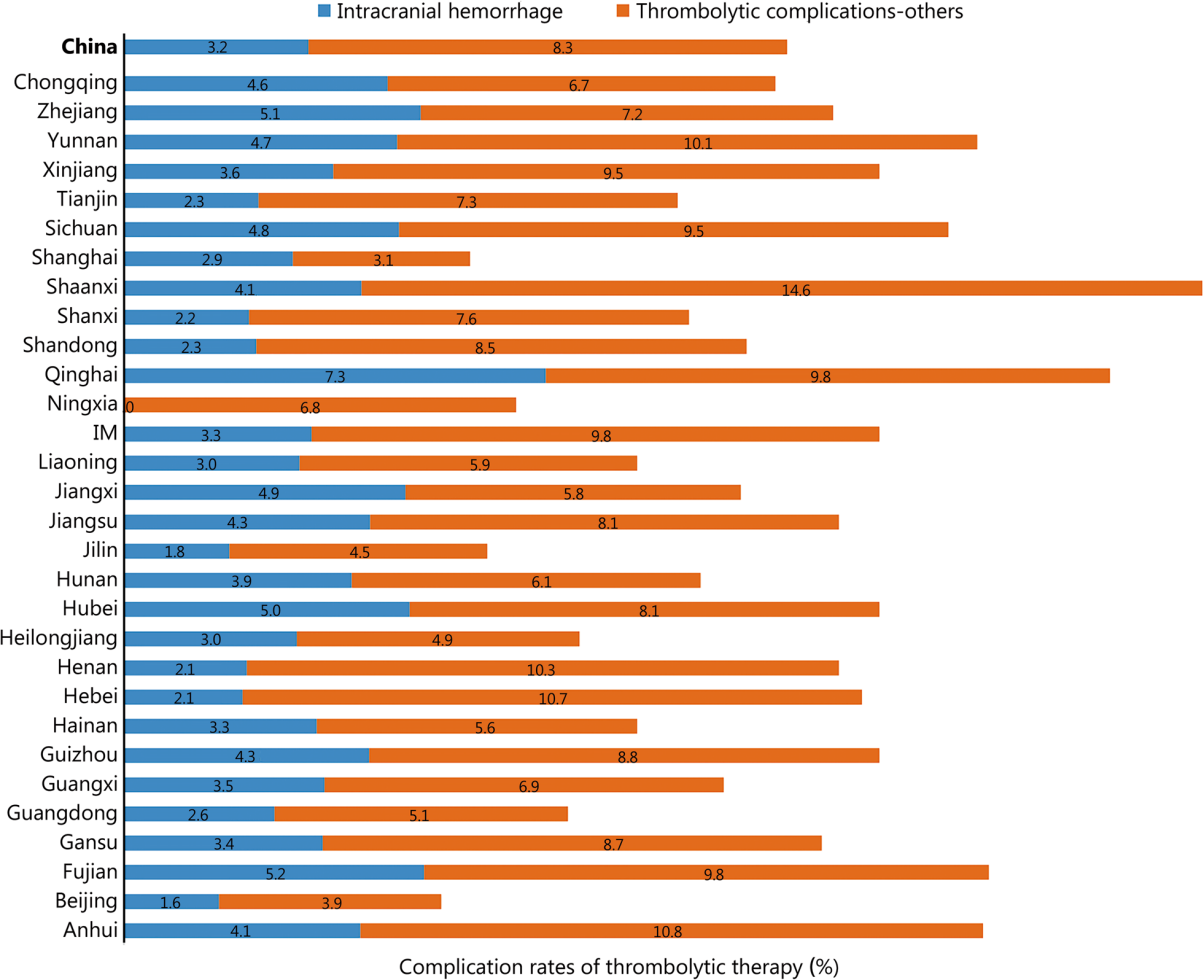
Complication rates of thrombolytic therapy in different provinces, 2019–2020 (%). Missing data from Xinjiang Production and Construction Corps and Tibet. IM Inner Mongolia

**Fig. 3:**
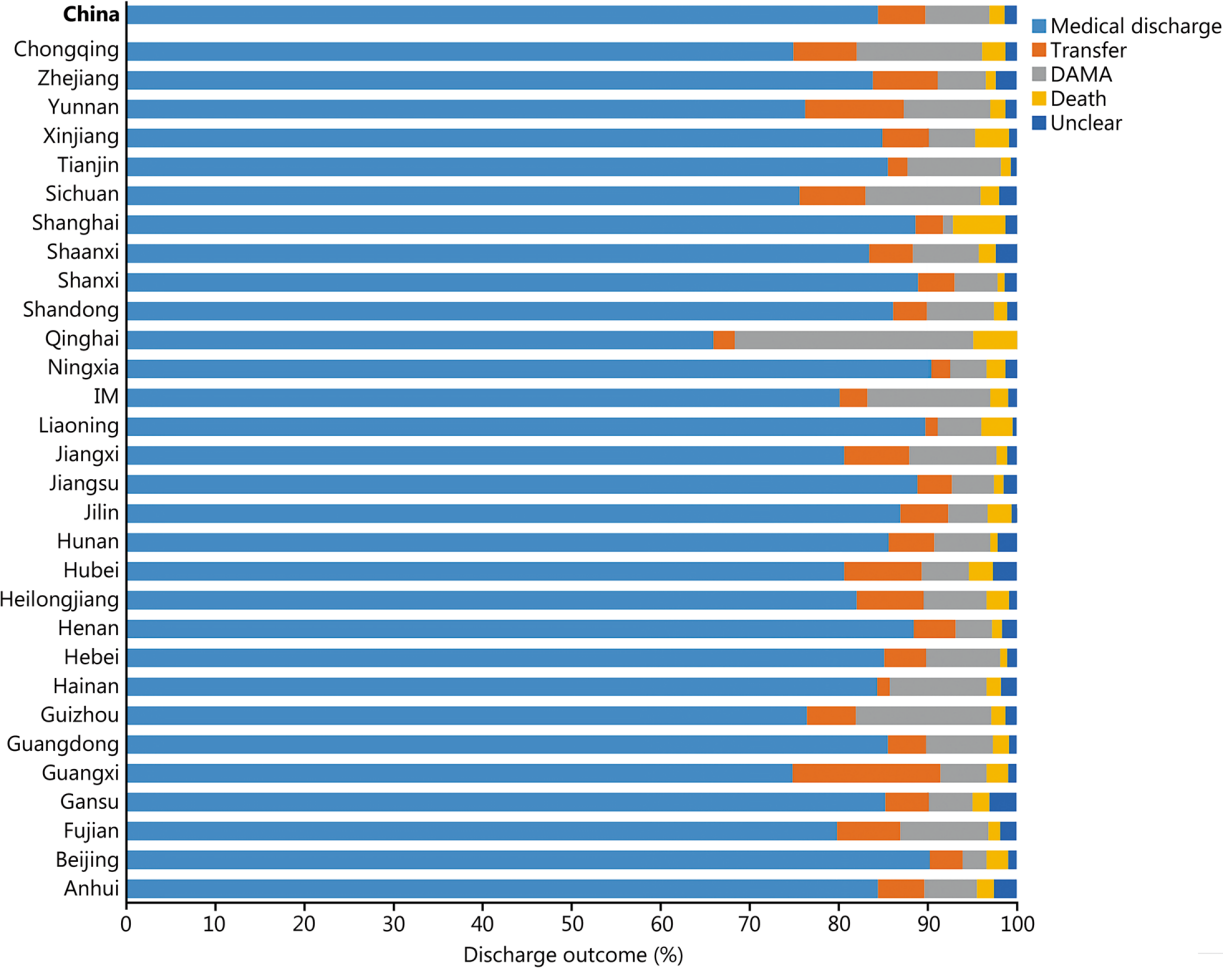
Discharge outcomes of patients receiving thrombolysis in different provinces, 2019–2020 (%). Missing data from Xinjiang Production and Construction Corps and Tibet. IM Inner Mongolia, DAMA discharge without medical advice

During the study period, the number of all acute IS patients was determined by the HQMS system. The thrombolysis rate is equal to thrombolysis patients/all acute IS patients × 100% (Additional file 2: Table S1). The national thrombolysis rate from 2019 to 2020 was 3.2% (95% CI 3.2–3.3%), ranging from 0.5% (95% CI 0.3–0.5%) in Qinghai and 8.3% (95% CI 8.1–8.3%) in Tianjin (Fig. 1). The rate of intracranial hemorrhage with thrombolytic therapy was 3.2% (95% CI 3.1–3.3%), ranging from 1.6 (95% CI 1.1–2.2%) in Beijing and 7.3% (95% CI -0.7 to 15.3%) in Qinghai (Fig. 2). In-hospital mortality with thrombolytic therapy was 1.7% (95% CI 1.7–1.8%), ranging from 0.8% (95% CI 0.8–0.9%) in Hebei, 0.8% (95% CI 0.8–1.1%) in Hunan and 0.8% (95% CI 0.8–1.0%) in Shanxi to 5.9% (95% CI 5.4–6.4%) in Shanghai. In-hospital death/discharge against medical advice rate was 8.9% (95% CI 8.9–9.0%), ranging from 5.2% (95% CI 4.9–5.5%) in Henan to 31.7% (95% CI 18.2–51.9%) in Qinghai (Fig. 3).

**Fig. 4:**
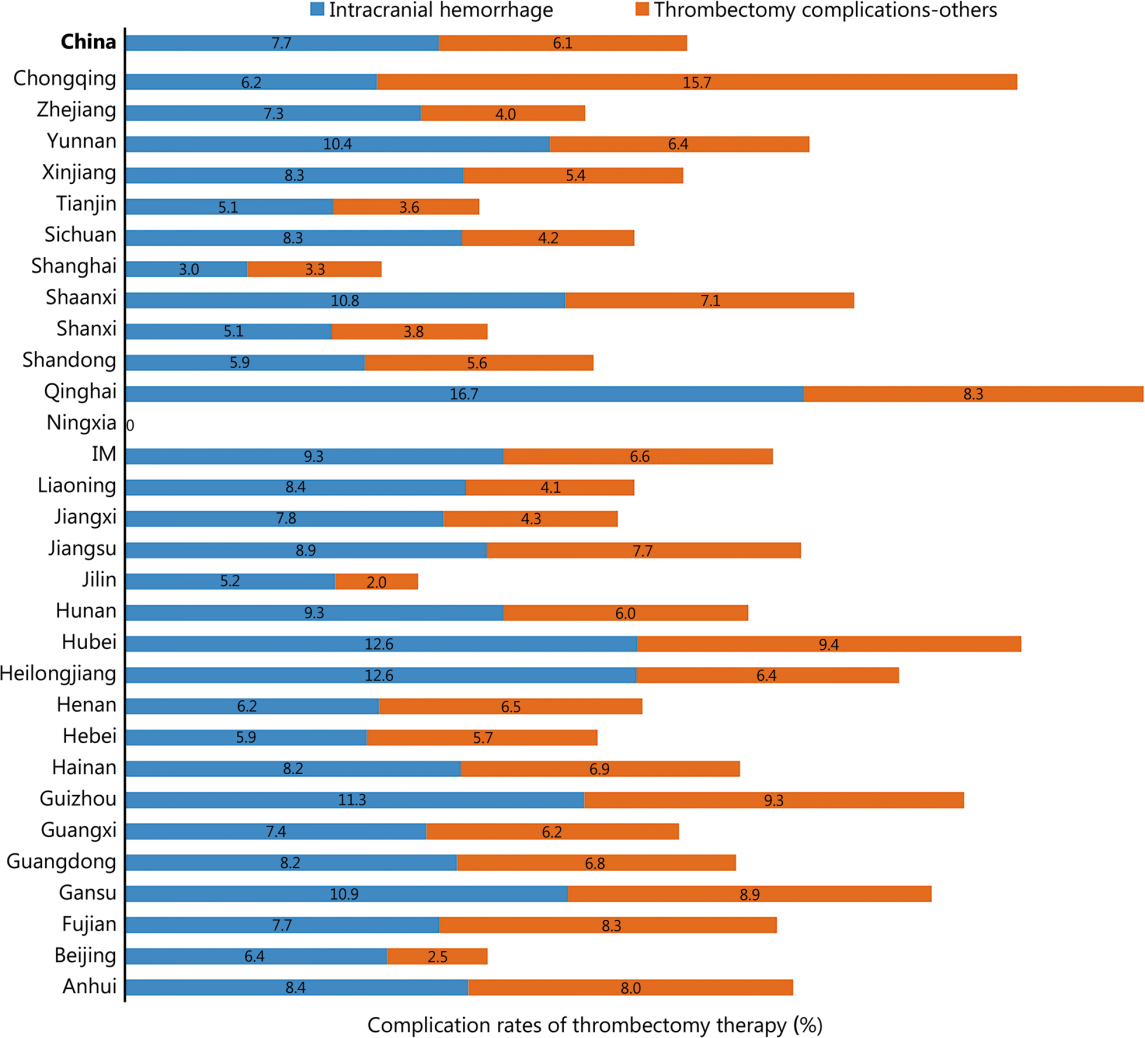
Complication rates of thrombectomy therapy in different provinces, 2019–2020 (%). Missing data from Xinjiang Production and Construction Corps and Tibet. IM Inner Mongolia

**Fig. 5:**
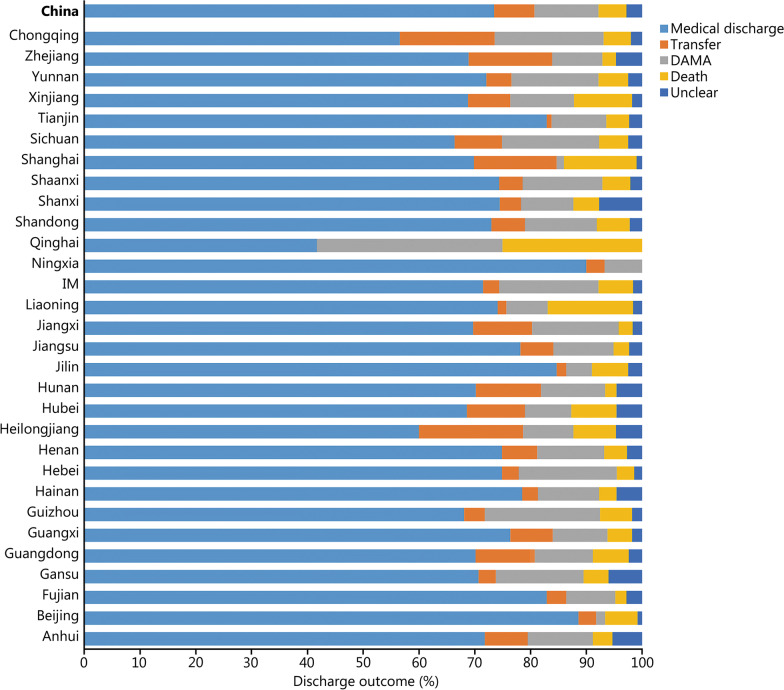
Discharge outcomes of patients with thrombectomy in different provinces, 2019–2020 (%). Missing data from Xinjiang Production and Construction Corps and Tibet. IM Inner Mongolia, DAMA discharge without medical advice

Thrombectomy rate is equal to thrombectomy patients/all acute IS patients × 100% (Additional file 2: Table S2). The national thrombectomy rate from 2019 to 2020 was 0.8% (95% CI 0.8–0.8%), ranging from 0.1% (95% CI 0.1–0.1%) in Qinghai and Ningxia to 2.4% (95% CI 2.4–2.4%) in Fujian (Fig. 1). The rate of intracranial hemorrhage with thrombectomy therapy was 7.7% (95% CI 7.5–8.0%), ranging from 3.0% (95% CI 1.3–4.7%) in Shanghai and 16.7% (95% CI -4.4 to 37.8%) in Qinghai (Fig. 4). In-hospital mortality in patients treated with thrombectomy was 5.0% (95% CI 4.8–5.2%), ranging from 2.0% (95% CI 1.2–2.8%) in Hunan and 2.0% (95% CI 1.4–2.7%) in Fujian to 25.0% (95% CI 0.5–49.5%) in Qinghai. In-hospital death/discharge against medical advice rate was 16.5% (95% CI 16.0–17.0%), ranging from 6.7% (95% CI -2.3 to 15.6%) in Ningxia to 58.3% (95% CI 3.0–86.2%) in Qinghai (Fig. 5).

**Fig. 6:**
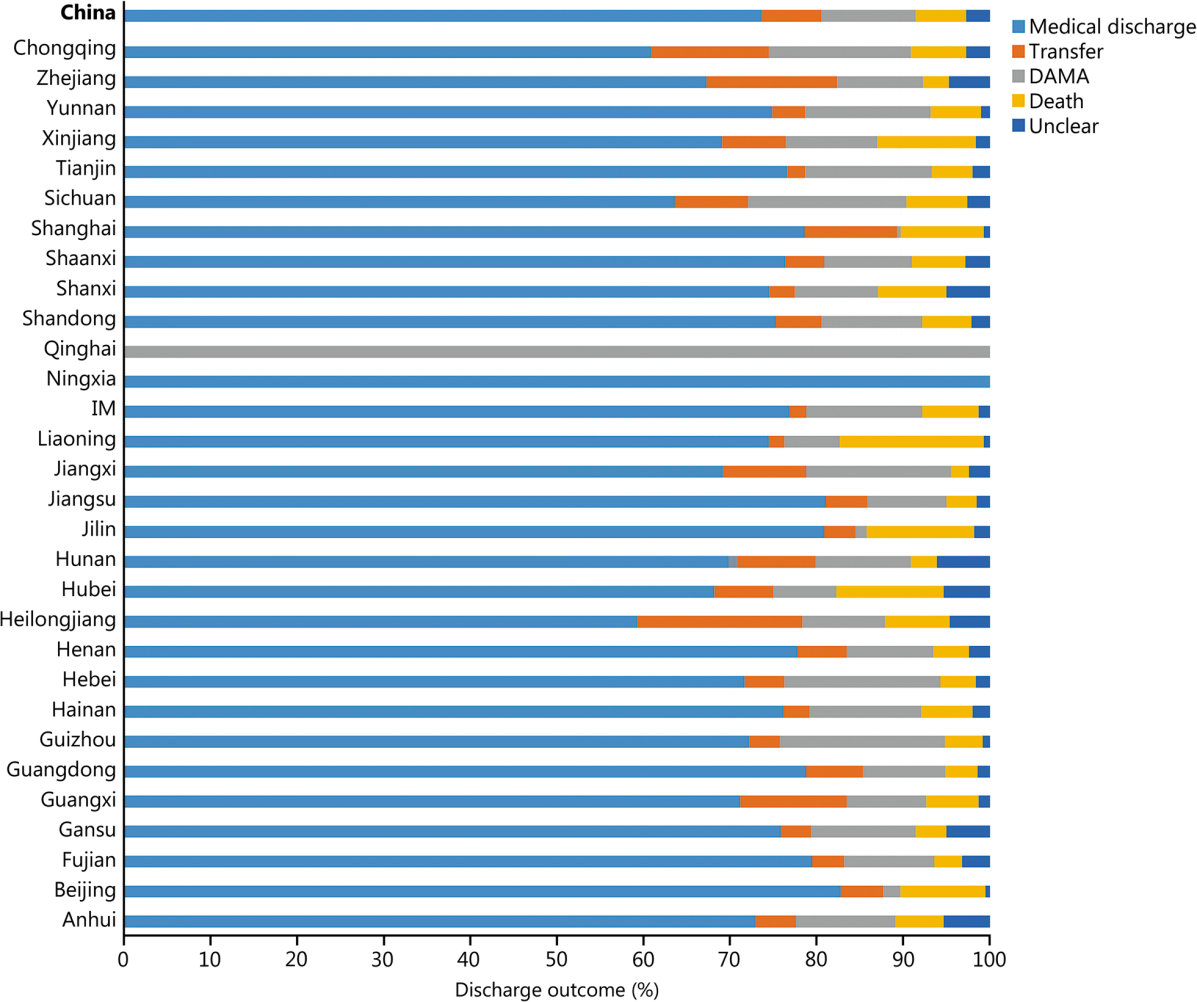
Discharge outcomes of patients receiving bridging therapy in different provinces, 2019–2020 (%). Missing data from Xinjiang Production and Construction Corps and Tibet. IM Inner Mongolia, DAMA discharge without medical advice

Bridging rate is equal to bridging patients/all acute ischemic stroke patients × 100% (Additional file 2: Table S3). The national bridging rate from 2019 to 2020 was 0.2% (95% CI 0.2–0.2%), ranging from 0% (95% CI 0.0–0.0%) in Qinghai and Ningxia to 0.5% (95% CI 0.5–0.5%) in Beijing, Fujian, Guangxi, Guizhou, Jiangxi, Xinjiang, Zhejiang, and Chongqing (Fig. 1 and Additional file 2: Table S3). The rate of intracranial hemorrhage with bridging therapy was 12.9% (95% CI 12.3–13.4%), ranging from 5.9% (95% CI 4.6–7.1%) in Guangdong and 26.7% (95% CI 23.2–30.3%) in Guangxi. In-hospital mortality in patients treated with bridging was 5.8% (95% CI 5.4–6.2%), ranging from 2.1% (95% CI 0.7–3.5%) in Jiangxi and 16.6% (95% CI 13.6–19.5%) in Liaoning. In-hospital death/discharge against medical advice rate was 16.8% (95% CI 15.9–17.7%), ranging from 10.0% (95% CI 5.4–14.7%) in Shanghai to 25.3% (95% CI 22.3–28.3%) in Sichuan (Fig. 6). The sample sizes of Qinghai and Ningxia were too small (*n* < 2) to be included in the statistics.

## BOSC-based Stroke Registry Study

A prospective nationwide hospital-based study was conducted at 231 stroke base hospitals (level III) from 31 provinces in China through BOSC. From January 2019 to December 2020, all patients with stroke {IS [international classification of diseases (ICD) 63], ICH (ICD61), and SAH (ICD60)} and with symptom onset within 14 d were included. A complete description of the information collected during admission and hospitalization has been reported previously [106–109]. In brief, demographic data, comorbidities and risk factors, stroke severity on admission, and acute treatment were recorded. After admission, all participants were followed up twice: 3 (telephone) and 12 months (face-to-face). Functional outcomes were assessed at follow-up by a mRS score (range from 0 to 6), wherein a good outcome was defined as an mRS of 0–2 points. Stroke recurrence and all-cause mortality were also recorded in the follow-up. Stroke recurrence was defined as suddenly deteriorated neurological function evaluated as a decreased NIH Stroke Scale score of 4 or more or a new focal neurological deficit of vascular origin that lasted for > 24 h [112].

Finally, 222 stroke base hospitals in 30 provinces (excluding Tibet) with effectively uploaded data were included. In total, 136,282 stroke patients [median (IQR) age, 68 (58–74) years; 61.2% male] were recruited and finished a 12-month follow-up. Of those, over 86.9% were IS [median (IQR) age, 68 (56–74) years; 61.3% male], 10.8% were ICH strokes [median (IQR) age, 63 (53–70) years; 63.6% male] and 2.3% were SAH strokes [median (IQR) age, 57 (51–67) years; 44.6% male]. Approximately 136,071 (87.7%) were first attacks, and 16,714 (12.3%) were recurrent attacks. In IS, 5.4% and 1.3% of patients received intravenous thrombolysis and endovascular therapy. An estimated 10.5% of ICH and 33.8% of SAH received craniotomy treatment, while 3.2% of ICH and 24.3% of SAH received stereotactic treatment. The in-hospital mortality rate for stroke was 1.3% (95% CI 1.2–1.4%). The length of stay [median (IQR)] for IS, ICH, and SAH was 12 (IQR 9–15) d, 13 (IQR 10–16) and 14 (IQR 10–17), respectively, while the in-hospital mortality rate [% (95% CI)] was 0.8% (95% CI 0.8–0.9%), 4.6% (95% CI 4.3–4.9%), and 4.4% (95% CI 3.9–5.1%). More information is listed in Table 3.

**Table 3:** Demographic characteristics and clinical information of the stroke patients included in BOSC-based Stroke Registry Study

Characteristics	IS (*n* = 118,425)	ICH (*n* = 14,708)	SAH (*n* = 3149)
Male [*n* (%)]	72,595 (61.3)	9354 (63.6)	1404 (44.6)
Age [years, median (IQR)]	68 (56–74)	63 (53–70)	57 (51–67)
Full expense [*n* (%)]	5684 (4.8)	1235 (8.4)	321 (10.2)
Patients with time from symptom onset to hospital arrival within 2 h [*n* (%)]	11,605 (9.8)	2427 (16.5)	636 (20.2)
Transported to hospital by EMS [*n* (%)]	8882 (7.5)	2868 (19.5)	712 (22.6)
Risk factors [*n* (%)]			
A history of stroke	14,211 (12.0)	2235 (15.2)	268 (8.5)
Hypertension	81,121 (68.5)	11,501 (78.2)	1587 (50.4)
DM	25,106 (21.2)	1691 (11.5)	334 (10.6)
AF	4500 (3.8)	221 (1.5)	28 (0.9)
TIA	13,619 (11.5)	1485 (10.1)	277 (8.8)
TOAST classification in ischemic stroke [*n* (%)]		–	–
1-LAA	51,515 (43.5)		
2-CE	13,619 (11.5)		
3-SVO	36,949 (31.2)		
4-SOE	5329 (4.5)		
5-SUE	11,014 (9.3)		
Severity at admission [median (IQR)]			
NIHSS or GCS score	3 (1–5)	12 (8–15)	13 (10–15)
mRS score	1 (1–3)	3 (1–4)	2 (1–4)
Intravenous thrombolysis [*n* (%)]	6394 (5.4)	–	–
DNT [min, median (IQR)]	44 (27–64)	–	–
ONT [min, median (IQR)]	166 (145–202)	–	–
Thrombolytic complications	908 (14.2)		
Intracranial hemorrhage	192 (3.0)		
Endovascular therapy [*n* (%)]	1539 (1.3)	–	–
Recanalization rate	1248 (81.1)		
Complications	228 (14.8)		
Intracranial hemorrhage	105 (6.8)		
Craniotomy treatment [*n* (%)]	–	1544 (10.5)	1064 (33.8)
Stereotactic treatment [*n* (%)]	–	471 (3.2)	765 (24.3)
Antiplatelet therapy for noncardiac stroke [*n* (%)]	82,564 (78.8) ^*^		
Swallowing function assessment rate [*n* (%)]	85,858 (72.5)	4457 (30.3)	929 (29.5)
Rehabilitation rate [*n* (%)]	71,529 (60.4)	7383 (50.2)	1622 (51.5)
Length of stay [d, median (IQR)]	12 (9–15)	13 (10–16)	14 (10–17)
Hospitalization costs/patient [CNY, median (IQR)]	16,004 (7484–19,767)	18,439 (9898–34,567)	79,598 (19,726–142,085)
Severity at discharge-mRS score [median (IQR)]	1 (0–2)	2 (1–4)	1 (0–3)
In-hospital mortality {*n* [ % (95% CI)]}	947 [0.8 (0.8–0.9)]	677 [4.6 (4.3–4.9)]	139 [4.4 (3.9–5.1)]

^*^n = 104,806

*IS* ischemic stroke, *ICH* intracerebral hemorrhage, *SAH* subarachnoid hemorrhage, *IQR* interquartile range, *BMI* body mass index, *EMS* Emergency Medical Services, *LAA* large artery atherosclerosis, *SVO* small vessel occlusion, *CE* cardioembolism, *SOE* stroke of other determined etiology, *SUE* stroke of undetermined etiology, *NIHSS* NIH Stroke Scale score, *DNT* Door to Needle Time, *ONT* Onset to Needle Time, *CNY* Chinese Yuan, *GCS* Glasgow Coma Scale, *mRS* modified Rankin Scale, *TIA* transient ischemic attack, *DM* diabetes mellitus, *AF* atrial fibrillation

The mRS scores at admission, discharge, 3-month and 12-month had been obtained and presented in the Tables 3–4 and Additional file 1: Figs. S10–12. The mRS score at discharge [median (IQR)] for IS, ICH, and SAH was 1 (IQR 0–2), 2 (IQR 1–4) and 1 (IQR 0–3), respectively. The disability rate [% (95% CI)] in survivors of stroke at 3-month and 12-month was 14.8% (95% CI 14.6–15.0%) and 14.0% (95% CI 13.8–14.2%), respectively. At 3-month follow-up, the disability rate [% (95% CI)] in survivors for IS, ICH, and SAH was 13.0% (95% CI 12.8–13.2%), 28.6% (95% CI 27.8–29.4%), and 23.1% (95% CI 21.6–24.7%), respectively. At the 12-month follow-up, these data were 12.4% (95% CI 12.2–12.6%), 29.1% (95% CI 28.3–29.9%), 12.6% (95% CI 11.4–13.8%), respectively. The mortality rate [% (95% CI)] of stroke at 3-month and 12-month was 4.2% (95% CI 4.1–4.3%) and 8.5% (95% CI 8.4–8.6%), respectively. The 3-month mortality rate for IS, ICH, and SAH was 3.2% (95% CI 3.1–3.3%), 11.2% (95% CI 10.7–11.7%), and 8.9% (95% CI 7.9–9.9%), while the 12-month mortality rate was 7.2% (95% CI 7.1–7.3%), 17.9% (95% CI 17.3–18.5%), and 13.5% (95% CI 12.3–14.7%). The recurrence rate [% (95% CI)] of stroke at 3-month and 12-month was 3.6% (95% CI 3.5–3.7%) and 5.6% (95% CI 5.4–5.7%), respectively. The 3-month stroke recurrence rate for IS, ICH, and SAH was 3.8% (95% CI 3.7–3.9%), 2.0% (95% CI 2.0–2.1%), and 1.9% (95% CI 1.5–2.4%), while 12-month stroke recurrence rate was 5.8% (95% CI 5.7–6.0%), 3.8% (95% CI 3.7–3.9%), and 3.3% (95% CI 2.7–4.0%) (Table 4).

**Table 4:** Stroke follow-up information

Characteristics	IS (*n* = 118,425)	ICH (*n* = 14,708)	SAH (*n* = 3149)
3-Month mRS score [*n*(%)]^†^			
0	44,527 (37.6)	3662 (24.9)	982 (31.2)
1	35,764 (30.2)	3133 (21.3)	803 (25.5)
2	19,421 (16.4)	2530 (17.2)	419 (13.3)
3	9237 (7.8)	1824 (12.4)	299 (9.5)
4	3434 (2.9)	1191 (8.1)	183 (5.8)
5	2250 (1.9)	721 (4.9)	183 (5.8)
6	3790 (3.2)	1647 (11.2)	280 (8.9)
3-Month disability rate in survivors {*n* [% (95% CI)]}	14,922 [13.0 (12.8–13.2)]*	3736 [28.6 (27.8–29.4)]**	664 [23.1 (21.6–24.7)]***
3-Month death {*n* [% (95% CI)]}	3790 [3.2 (3.1–3.3)]	1647 [11.2 (10.7–11.7)]	280 [8.9 (7.9–9.9)]
3-Month recurrence events {*n* [% (95% CI)]}	4523 [3.8 (3.7–3.9)]	301 [2.0 (2.0–2.1)]	61 [1.9 (1.5–2.4)]
12-Month mRS score [*n* (%)]			
0	49,857 (42.1)	3412 (23.2)	1263 (40.1)
1	33,277 (28.1)	2721 (18.5)	715 (22.7)
2	13,145 (11.1)	2427 (16.5)	403 (12.8)
3	7698 (6.5)	1912 (13)	186 (5.9)
4	4619 (3.9)	897 (6.1)	94 (3)
5	1303 (1.1)	706 (4.8)	63 (2)
6	8526 (7.2)	2633 (17.9)	425 (13.5)
12-Month disability rate in survivors {*n* [% (95% CI)]}	13,619 [12.4 (12.2–12.6)]^#^	3515 [29.1 (28.3–29.9)]^##^	343 [12.6 (11.4–13.8)]^###^
12-Month death {*n* [% (95% CI)]}	8527 [7.2 (7.1–7.3)]	2633 [17.9 (17.3–18.5)]	425 [13.5 (12.3–14.7)]
12-Month recurrence events {*n* [% (95% CI)]}	6912 [5.8 (5.7–6.0)]	558 [3.8 (3.7–3.9)]	105 [3.3 (2.7–4.0)]

^†^Two patients’ scores were missing. ^*^n = 114,635, ^**^n = 13,061, ^***^n = 2826, ^#^*n* = 109,899, ^##^*n* = 12,075, ^###^*n* = 2723. *IS* ischemic stroke, *ICH* intracerebral hemorrhage, *SAH* subarachnoid hemorrhage, *mRS* modified Rankin Scale

## Prospects for the future work of stroke prevention and control in China

2021 is the first year of the “14th Five-Year Plan”. The CSPPC will actively implement the “Comprehensive Program for Strengthening Stroke Prevention and Control to Reduce Million New Disability Projects” and promote China’s stroke prevention and control work to a new level. CSPPC will focus on “Stroke Recognition Action in Thousands of Counties and Ten Thousand of Towns” and “Stroke Graded Diagnosis and Treatment Action” to implement the goal of the “Million Disabled Reduction Project”. Through the implementation of actions, the general public’s ability to identify stroke in the early stage will be improved, the power of grassroots stroke identification and scientific transport will be strengthened, and the level of stroke diagnosis and treatment in relevant medical institutions at all levels in the region will be improved, the life-cycle health management of stroke patients will be carried out, and two-way referrals will be implemented.

The CSPPC aims to enhance the prevention and treatment capabilities of county-level stroke centers, consolidate the progress made in their establishment, integrate the construction of stroke centers into the assessment criteria of local health departments, and expedite the development of stroke centers in county hospitals. By 2022, in all counties with more than 300,000 people, at least one secondary general hospital can routinely carry out thrombolysis technology. Continuing with on-site guidance and training in stroke centers, an expert group has been assembled to provide on-site advice and training for each unit of stroke centers. This guidance follows nationally standardized assessment criteria, enabling the group to offer suggestions for enhancing the hospital’s stroke management model, processes, and diagnosis and treatment technologies.

By leveraging the BOSC, the CSPPC proactive efforts are made to carry out national unified and standardized stroke diagnosis and treatment case registration management. Additionally, there is an exploration of information interconnection and secure sharing among medical and health institutions within the region. This approach involves gradually implementing screening and registration for stroke risk factors, emergency stroke treatment, rehabilitation follow-up, and dynamic management of death information for the targeted population. These initiatives aim to enhance the efficiency of national stroke prevention and control efforts. Furthermore, the collected data are transformed into academic scientific research outcomes, providing a solid foundation for the cardiovascular and cerebrovascular disease prevention and control actions outlined in the “Healthy China 2030” strategy.

The CSPPC is committed to expediting the training a cadre of highly skilled and proficient personnel in stroke prevention and control. This includes strengthening technical training in thrombolysis, thrombectomy, and vascular ultrasound. Moreover, there is a focus on enhancing the training of first aid personnel in stroke recognition and transportation, improving their capacity to provide immediate assistance. To ensure comprehensive coverage, assistance activities will be conducted in ethnic minority areas in the central and Western regions and at the grassroots level. This will involve establishing relevant incentive systems, promoting collaborative assistance between eastern and central provinces, and targeting aid in Western ethnic regions. The ultimate goal is to enhance stroke diagnosis, treatment, and management standardization.

The CSSPC also launch Healthy China 2030 Stroke Action Plan (Table 5). In summary, the target is to 1) reduce the stroke incidence (mortality) to 290 (200)/100,000 person-years by 2025, and to 270 (190) by 2030; 2) increase the control rates of hypertension and diabetes in stroke survivors to 38% and 61% by 2025, and 42% and 65% by 2030; 3) increase the rates of thrombolysis and thrombectomy in acute IS to 5.0% and 1.2% by 2025, and 8.0% and 1.6% by 2030; 4) reduce the mortality rates of thrombolysis and thrombectomy to 1.5% and 4.2% by 2025, and 1.2% and 3.5% by 2030; 5) reduce the percentage of out-of-pocket payment in total health expenditure to 30% by 2025, and to 28% by 2030; 6) increase the rehabilitation rate of hospitalized stroke patients to 40% by 2025, and 50% by 2030; 7) increase the medical referral rate for discharged stroke patients to 5.0% by 2025, and 7.5% by 2030; 8) increase the death/discharge against medical advice rate for discharged stroke patients to 6.5% by 2025, and 4.8% by 2030; 9) reduce 3-month mortality in stroke patients to 3.5% by 2025, and 3.0% by 2030; 10) reduce 3-month disability rate in stroke survivors to 13.5% by 2025, and 12.0% by 2030; 11) reduce 12-month mortality in stroke patients to 7.5% by 2025, and 6.5% by 2030; 12) reduce 12-month disability rate in stroke survivors to 11.5% by 2025, and 10.5% by 2030; 13) increase the 3-month and 12-month outpatient follow-up rates for stroke patients to 20% and 30% by 2025, and 30% and 40% by 2030; 14) increase the 3-month and 12-month professional rehabilitation rates for stroke patients to 30% and 40% by 2025, and 40% and 50% by 2030. This plan aims to practice the goal of Healthy China, improve health services and the health industry, promote the overall management of stroke prevention, treatment and rehabilitation, reduce stroke morbidity, disability and mortality, improve the quality of life of Chinese residents, and reach the leading health indicators of high-income countries by 2030.

**Table 5:** Stroke prevention and treatment goals in 2030

Characteristics	Stroke			IS			ICH			SAH		
	2020	2025	2030	2020	2025	2030	2020	2025	2030	2020	2025	2030
Epidemiological characteristics of stroke^‡^				–	–	–	–	–	–	–	–	–
Prevalence (%)	1.6	1.7	1.8									
Incidence/100,000 person–years	310.0	290.0	270.0									
Mortality/100,000 person–years	210.0	200.0	190.0									
Risk factors in stroke patients (%)				–	–	–	–	–	–	–	–	–
Obesity^††^	18.5	17.0	15.0									
Smoking	16.0	14.0	12.0									
Drinking	16.0	14.0	12.0									
Physical inactivity	30.0	27.0	24.0									
Hypertension control rate	35.0	38.0	42.0									
Diabetes control rate	58.0	61.0	65.0									
Hyperlipidemia control rate	34.0	37.0	40.0									
Pre-hospital management (%)												
EMS	10.5	13.0	15.0	7.5	10.0	12.5	19.5	22.5	25.0	22.5	25.0	17.5
Acute treatment (%)												
Thrombolysis rate^†^				3.2	5.0	8.0						
Intracranial hemorrhage rate				3.2	2.9	2.5						
In-hospital mortality				1.7	1.5	1.2						
Thrombectomy rate^†^				0.8	1.2	1.6						
Intracranial hemorrhage rate				7.7	6.5	5.0						
In-hospital mortality				5.0	4.2	3.5						
Bridging rate^†^				0.2	0.5	0.8						
Bridging bleeding rate				12.9	10.5	9.0						
In-hospital mortality				5.8	5.0	4.0						
Serious postoperative complications							8.5	7.5	6.5	8.5	7.5	6.5
Rehabilitation rates during hospitalization	25.0	40.0	50.0	25.0	40.0	50.0	25.0	40.0	50.0	25.0	40.0	50.0
Length of stay (d, median)	10.7	10.0	9.5	10.5	10.0	9.0	17.2	16.0	15.0	14.7	13.5	12.5
Out-of-pocket expenses rate	34.1	30.0	28.0	33.4	30.0	28.0	33.3	30.0	28.0	37.8	30.0	28.0
Discharge outcome												
Medical transfer	2.5	5.0	7.5	2.5	4.0	6.0	3.6	5.0	7.5	4.9	7.5	10.0
Discharge against medical advice	7.1	5.5	4.0	5.7	5.0	4.0	17.2	15.0	12.5	17.0	15.0	12.5
Death	1.1	1.0	0.8	0.7	0.6	0.5	4.6	4.0	3.0	4.4	3.6	2.5
Post-discharge patient management at 3-month (%)												
Outpatient follow-up rate		20.0	30.0		20.0	30.0		20.0	30.0		20.0	30.0
Outpatient follow-up appointment rate		25.0	50.0		25.0	50.0		25.0	50.0		25.0	50.0
Professional rehabilitation rate		30.0	40.0		30.0	40.0		30.0	40.0		30.0	40.0
Social assistance rate		10.0	20.0		10.0	20.0		10.0	20.0		10.0	20.0
Stroke mortality	4.0	3.5	3.0	2.4	2.2	2.0	6.6	6.0	5.5	4.5	4.0	3.0
Disability rate in survivors	14.8	13.5	12.0	13.0	12.0	11.0	28.6	27.0	25.0	23.1	22.0	20.5
Stroke recurrence rate	3.2	2.9	2.5	3.8	3.5	3.2	2.0	1.8	1.6	1.9	1.7	1.5
Post-discharge patient management at 12-month (%)												
Outpatient follow-up rate		30.0	40.0		30.0	40.0		30.0	40.0		30.0	40.0
Outpatient follow-up appointment rate		25.0	50.0		25.0	50.0		25.0	50.0		25.0	50.0
Professional rehabilitation rate		40.0	50.0		40.0	50.0		40.0	50.0		40.0	50.0
Social assistance rate		20.0	30.0		20.0	30.0		20.0	30.0		20.0	30.0
Stroke mortality	8.5	7.5	6.5	7.0	6.0	5.0	18.0	16.5	15.0	14.0	13.0	12.0
Disability rate in survivors	12.5	11.5	10.5	12.4	11.5	10.5	29.0	17.0	25.0	12.5	11.5	10.5
Stroke recurrence rate	5.5	5.0	4.5	6	5.5	5.0	3.5	3.0	2.5	3.5	3	2.5

^‡^Chinese adult population ≥ 18 years of age. ^†^Patients treated in the acute phase/all hospitalized patients with acute ischemic stroke × 100%. ^††^Obesity was defined as body mass index greater than or equal to 28.0 kg/m^2^

*IS* ischemic stroke, *ICH* intracerebral hemorrhage, *SAH* subarachnoid hemorrhage, *EMS* Emergency Medical Services

The burden of stroke continues to increase due to an aging population, increased incidence of stroke risk factors such as hypertension, and inadequate control [7, 113, 114]. In fact, 10 potentially modifiable risk factors are collectively associated with about 90% of the PAR of stroke [22]. The CSPPC needs to further strengthen the control of controllable risk factors for stroke in the community, and improve the management efficiency of patients with chronic diseases. At present, China has made great efforts in stroke prevention, acute treatment, and chronic rehabilitation, but the current social burden caused by strokes in China, such as rehabilitation costs and labor losses, has not been studied much. In the next work plan, CSPPC will make up for these deficiencies. Relying on the “Reduction Million New Disability Project”, CSPPC and the government organization will take measures to reduce the disability and the living burden of the disabled family after stroke. The Chinese Stroke Association published the China Stroke Report 2021 and reported pre-existing conditions contributing to stroke according to large-scale epidemiological surveys and stroke inpatient information and outcomes according to HQMS [111]. In addition to the aforementioned areas, our surveillance report also presents data on community-based screening of high-risk stroke populations, acute stroke treatments (such as thrombolysis and thrombectomy), and post-discharge follow-up. We recognize the importance of gender equity in stroke prevention and treatment and will provide a detailed analysis of gender differences in our next annual report.

## Conclusions

The burden of stroke disease in China is on the rise due to an aging population and an increasing prevalence of stroke-related risk factors like diabetes and hypertension, which are inadequately controlled. Additionally, significant regional differences in stroke prevalence (such as the stroke belt) and treatment levels (with higher levels in the Eastern coastal areas compared to the underdeveloped Western areas) pose significant challenges to stroke prevention and control in China. This report establishes a database of community and stroke patients in China, providing a scientific basis for understanding the burden, epidemiological characteristics, treatment, and prognosis of stroke in China. These data can inform the development of effective stroke prevention and treatment strategies and facilitate the allocation of medical resources in China in the next decade.

## Supplementary Information

**Additional file 1: Fig. S1.** Composition of stroke in different provinces. **Fig. S2.** Discharge outcomes of inpatient stroke patients in China, 2020. **Fig. S3.** Ratio of stroke hospitalization costs in different provinces to the national average. **Fig. S4.** Ratio of stroke out-of-pocket expenses in different provinces to the national average. **Fig. S5.** Ratio of stroke out-of-pocket expense rate in different provinces to the national average. **Fig. S6.** In-hospital outcomes of IS admitted to hospitals in Hospital Quality Monitoring System and Bigdata Observatory Platform for Stroke of China in 2020 by provinces. **Fig. S7.** In-hospital outcomes of ICH admitted to hospitals in Hospital Quality Monitoring System and Bigdata Observatory Platform for Stroke of China in 2020 by provinces. **Fig. S8.** In-hospital outcomes of SAH admitted to hospitals in Hospital Quality Monitoring System and Bigdata Observatory Platform for Stroke of China in 2020 by provinces. **Fig. S9.** Discharge outcomes of different treatments for acute ischemic stroke, 2019–2020. **Fig. S10.** mRS score of ischemic stroke patients at different time points. **Fig. S11.** mRS score of intracerebral hemorrhage patients at different time points. **Fig. S12.** mRS score of subarachnoid hemorrhage patients at different time points

**Additional file 2: Table S1.** Thrombolytic therapy for acute ischemic stroke in different provinces, 2019–2020. **Table S2.** Mechanical thrombectomy for acute ischemic stroke in different provinces, 2019–2020. **Table S3.** Bridging therapy for acute ischemic stroke in different provinces, 2019–2020

## Data Availability

Please contact the corresponding author (Pro. Wang) for the data request.

## Abbreviations

BMI: Body mass index
BOSC: Bigdata Observatory Platform for Stroke of China
CI: Confidence interval
CKB: China Kadoorie Biobank
CNY: Chinese Yuan
CRAF: China Registry of Atrial Fibrillation
CSPPC: China Stroke Prevention Project Committee
DALYs: Disability-adjusted life years
DNT: Door to Needle Time
GBD: Global Burden of Disease
Hhcy: Hyperhomocysteinemia
HCY: Homocysteine
HQMS: Hospital Quality Monitoring System
HR: Hazard ratio
ICH: Intracerebral hemorrhage
IS: Ischemic stroke
IVT: Intravenous thrombolytic therapy
LDL: Low-density lipoprotein cholesterol
mRS: Modified Rankin Scale
MT: Mechanical thrombectomy
PAR: Population attributable risks
PM: Particulate matter
SAH: Subarachnoid hemorrhage
SD: Standard deviation
SE: Standard error
