# Supplementary material for: China stroke surveillance report 2021
Source: Mil Med Res. 2023 Jul 19;10:33. doi: 10.1186/s40779-023-00463-x (PMC10355019; doi:10.1186/s40779-023-00463-x)
Supplement: Supplementary file 1 — Additional file 1: Fig. S1. Composition of stroke in different provinces. Fig. S2. Discharge outcomes of inpatient stroke patients in China, 2020. Fig. S3. Ratio of stroke hospitalization costs in different provinces to the national average. Fig. S4. Ratio of stroke out-of-pocket expenses in different provinces to the national average. Fig. S5. Ratio of stroke out-of-pocket expense rate in different provinces to the national average. Fig. S6. In-hospital outcomes of IS admitted to hospitals in Hospital Quality Monitoring System and Bigdata Observatory Platform for Stroke of China in 2020 by provinces. Fig. S7. In-hospital outcomes of ICH admitted to hospitals in Hospital Quality Monitoring System and Bigdata Observatory Platform for Stroke of China in 2020 by provinces. Fig. S8. In-hospital outcomes of SAH admitted to hospitals in Hospital Quality Monitoring System and Bigdata Observatory Platform for Stroke of China in 2020 by provinces. Fig. S9. Discharge outcomes of different treatments for acute ischemic stroke, 2019–2020. Fig. S10. mRS score of ischemic stroke patients at different time points. Fig. S11. mRS score of intracerebral hemorrhage patients at different time points. Fig. S12. mRS score of subarachnoid hemorrhage patients at different time points [file 40779_2023_463_MOESM1_ESM.pdf]

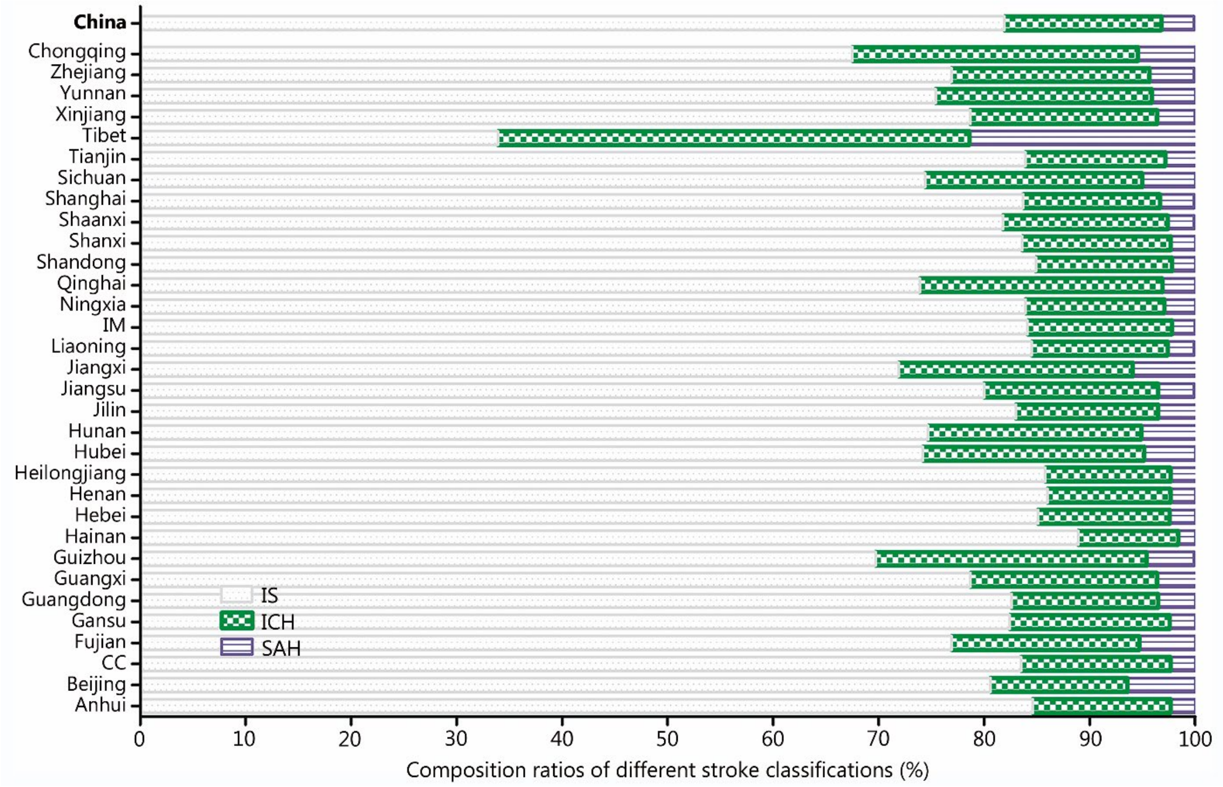

Fig. S1 Composition of stroke in different provinces. IS ischemic stroke, ICH intracerebral hemorrhage, SAH subarachnoid hemorrhage, CC Xinjiang Production and Construction Corps, IM Inner Mongolia

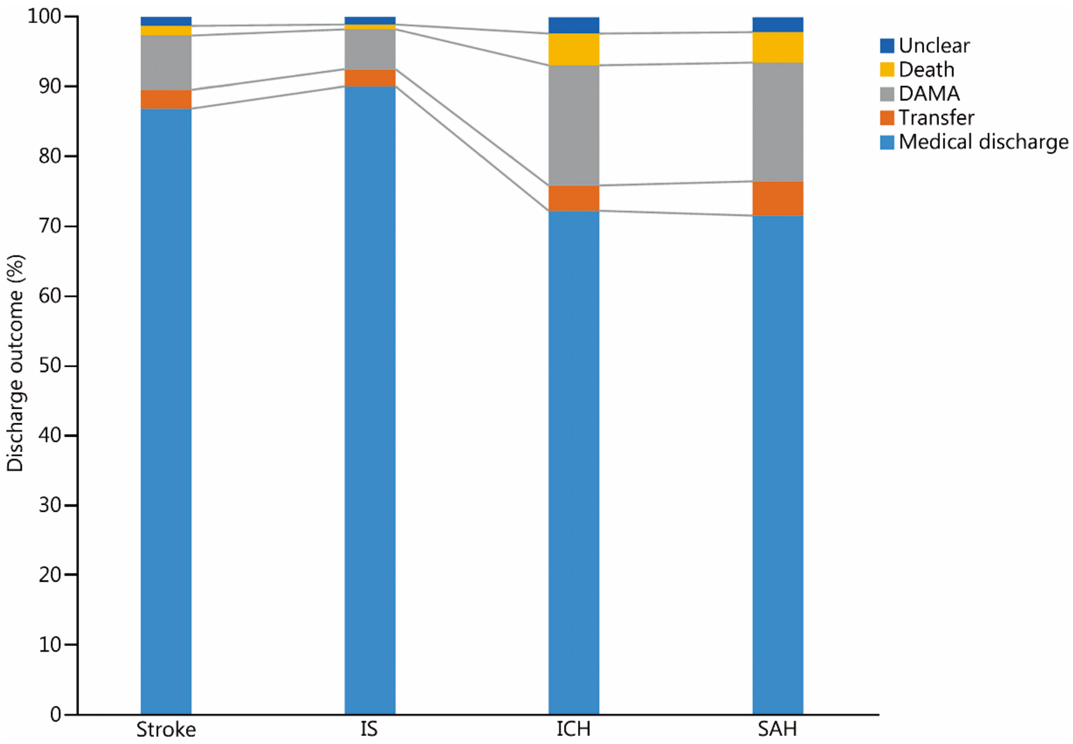

Fig. S2 Discharge outcomes of inpatient stroke patients in China, 2020 (%). IS ischemic stroke, ICH intracerebral hemorrhage, SAH, subarachnoid hemorrhage

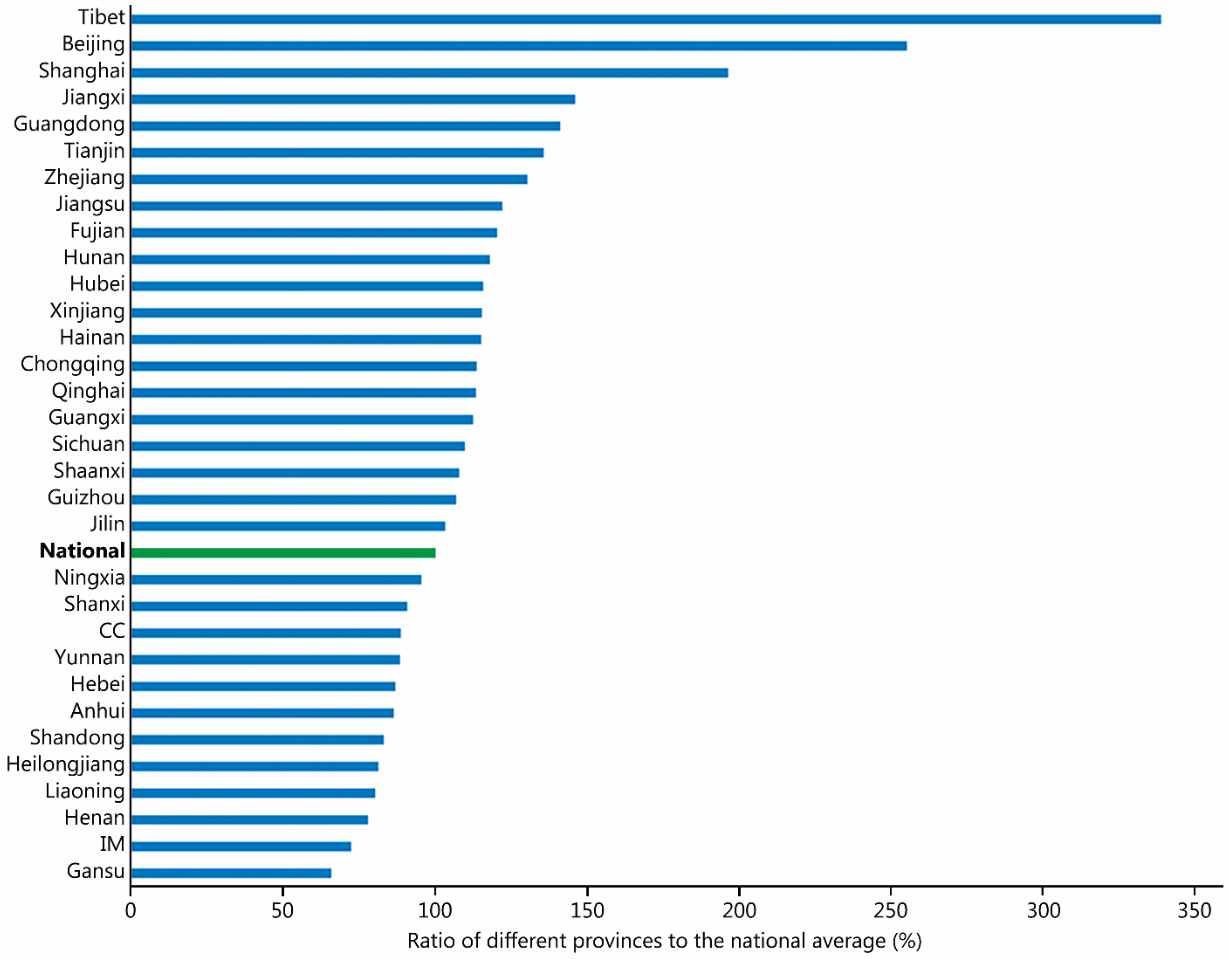

Fig. S3 Ratio of stroke hospitalization costs in different provinces to the national average (%). CC Xinjiang Production and Construction Corps, IM Inner Mongolia

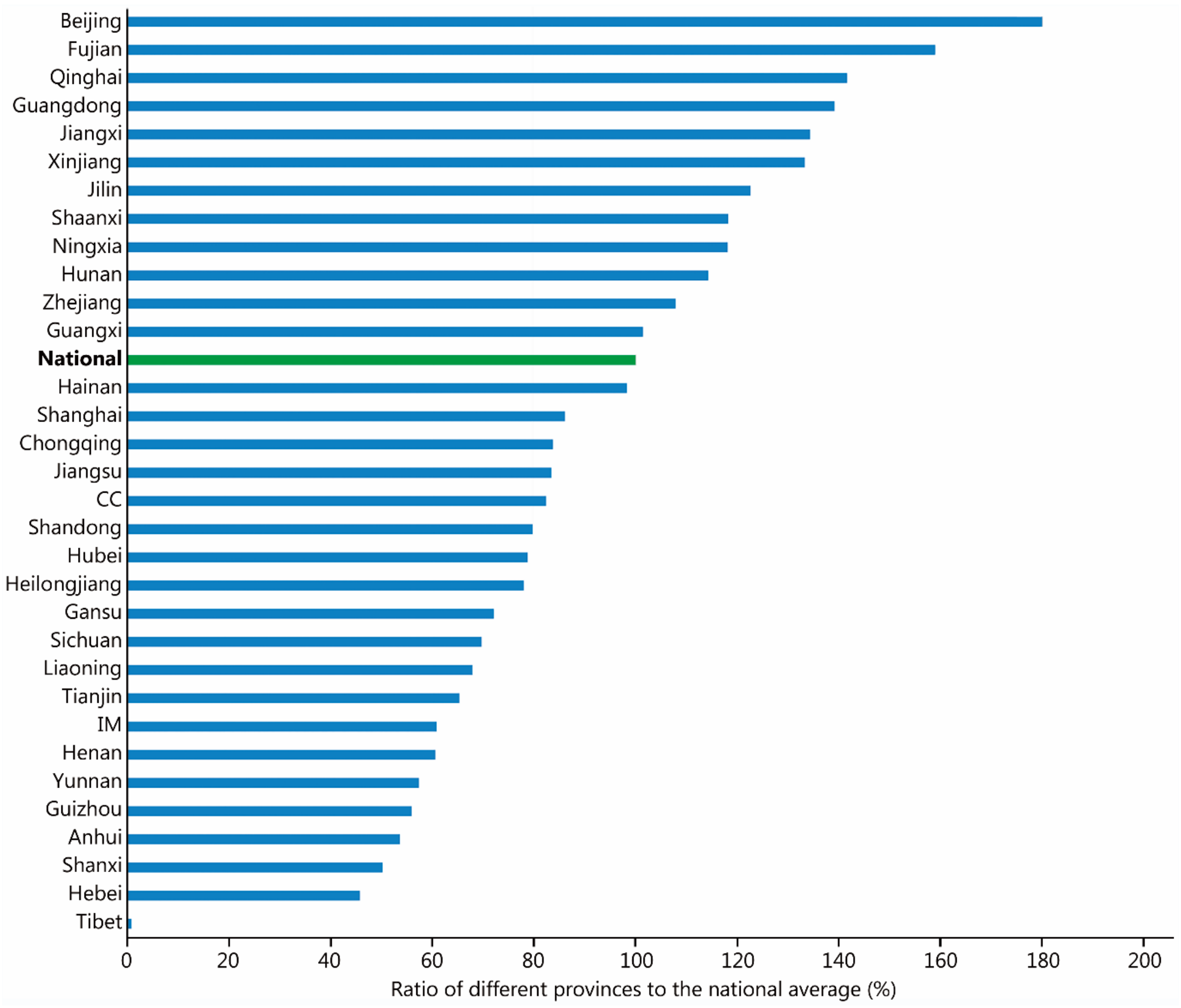

Fig. S4 Ratio of stroke out-of-pocket expenses in different provinces to the national average (%). CC Xinjiang Production and Construction Corps, IM Inner Mongolia

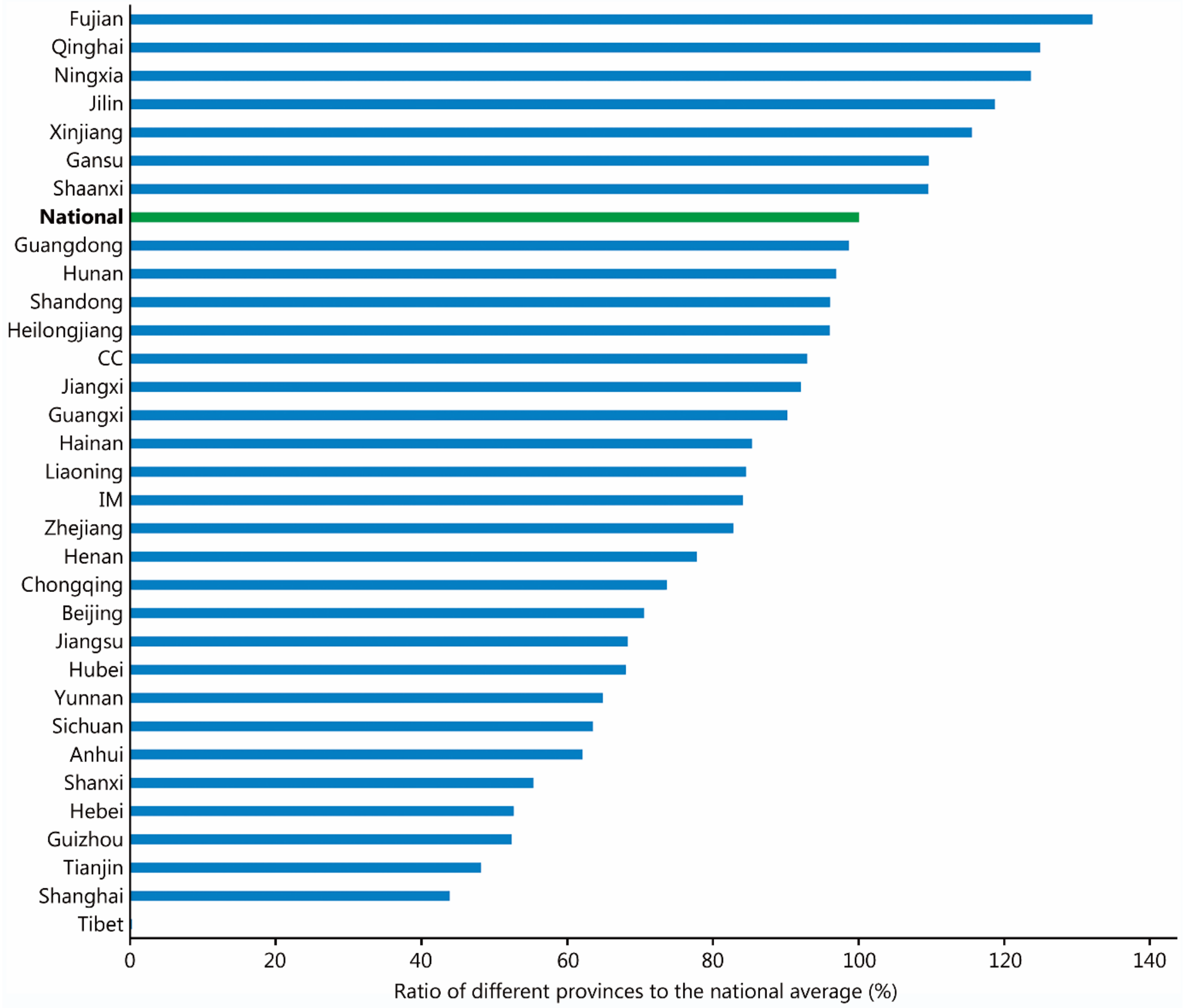

Fig. S5 Ratio of stroke out-of-pocket expense rate in different provinces to the national average (%). out-of-pocket expense rate = out-of-pocket expense/hospitalization expenditures. CC Xinjiang Production and Construction Corps, IM Inner Mongolia

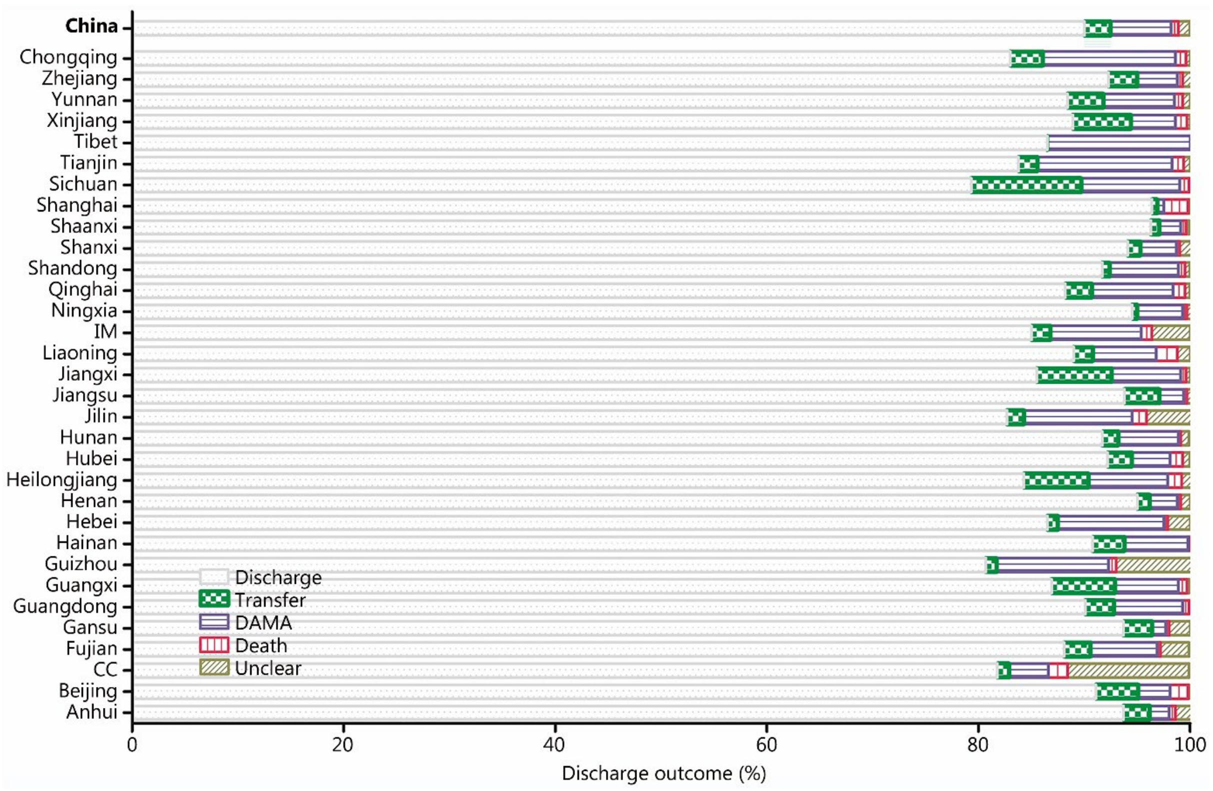

Fig. S6 In-hospital outcomes of IS admitted to hospitals in Hospital Quality Monitoring System and Bigdata Observatory Platform for Stroke of China in 2020 by provinces (%). DAMA discharge without medical advice, IS ischemic stroke, CC Xinjiang Production and Construction Corps, IM Inner Mongolia

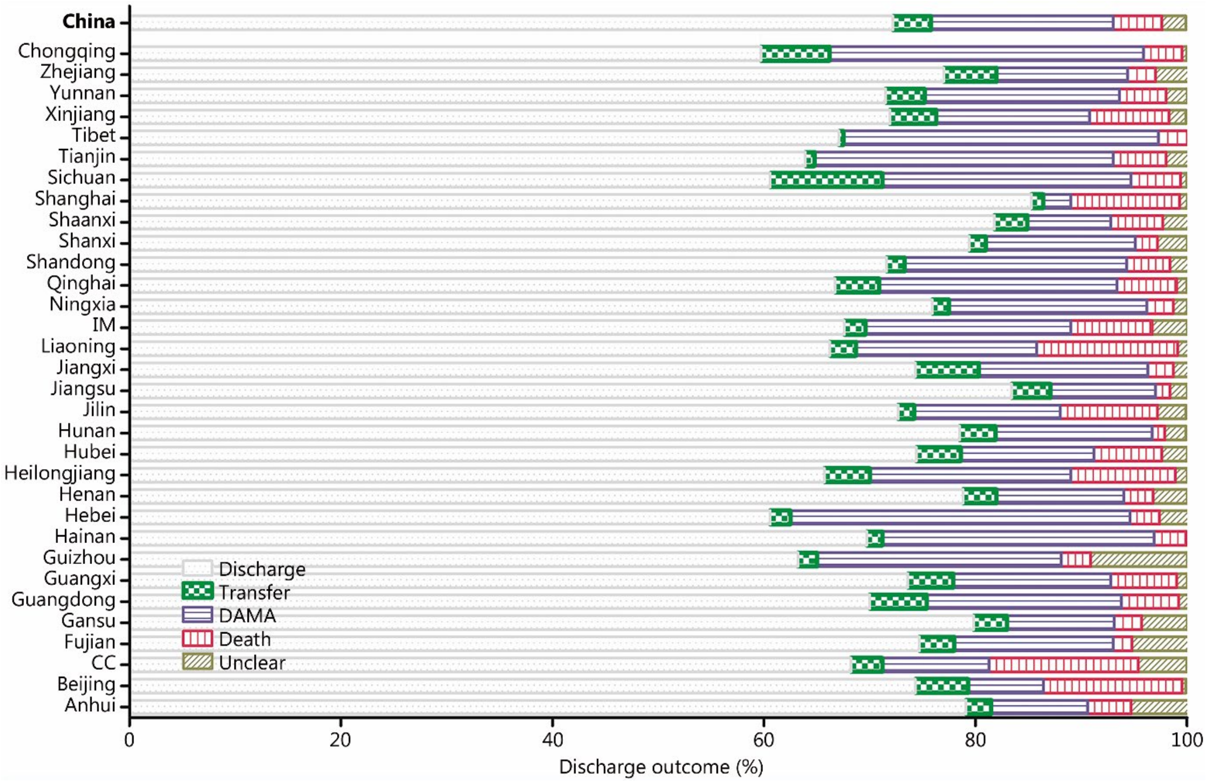

Fig. S7 In-hospital outcomes of ICH admitted to hospitals in Hospital Quality Monitoring System and Bigdata Observatory Platform for Stroke of China in 2020 by provinces (%). DAMA discharge without medical advice, ICH intracerebral hemorrhage, CC Xinjiang Production and Construction Corps, IM Inner Mongolia

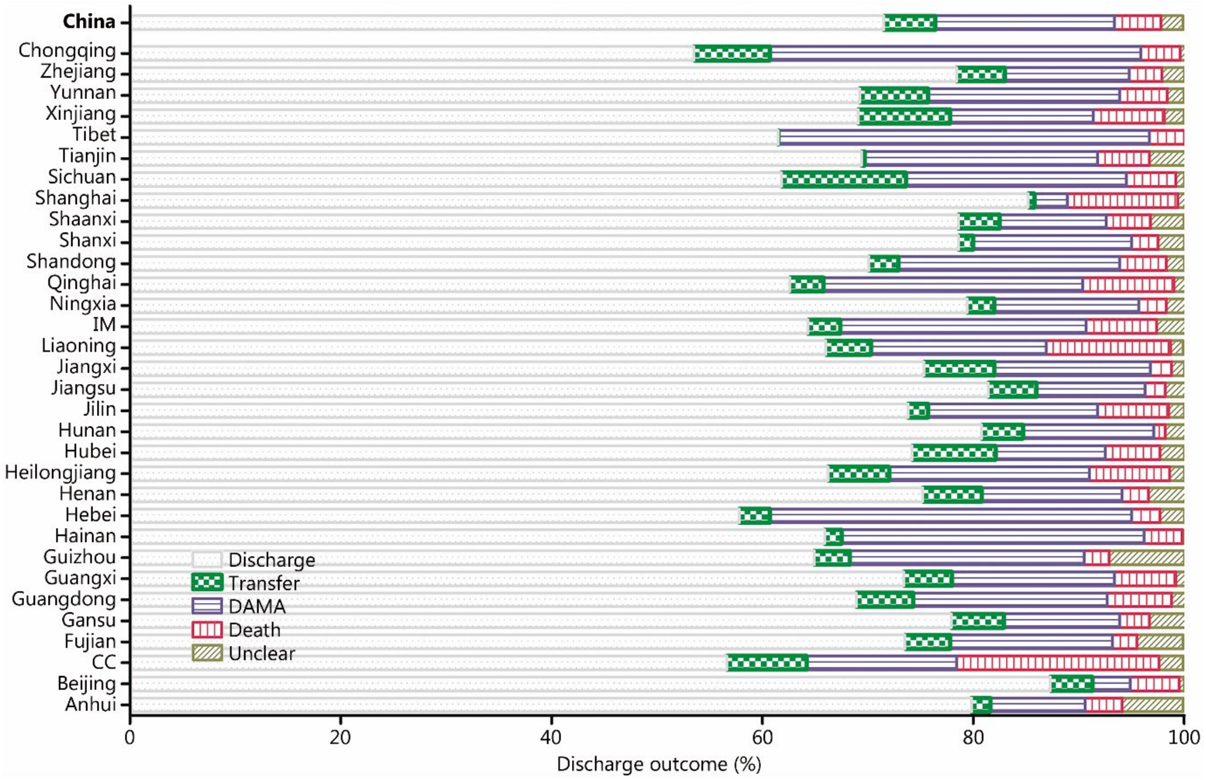

Fig. S8 In-hospital outcomes of SAH admitted to hospitals in Hospital Quality Monitoring System and Bigdata Observatory Platform for Stroke of China in 2020 by provinces (%). DAMA discharge without medical advice, SAH subarachnoid hemorrhage, CC Xinjiang Production and Construction Corps, IM Inner Mongolia

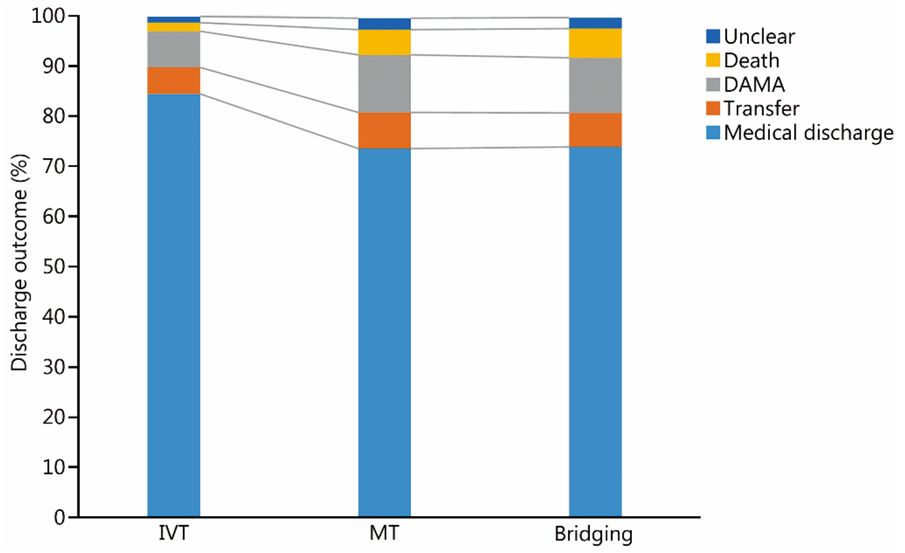

Fig. S9 Discharge outcomes of the patients receiving different treatments for acute ischemic stroke, 2019 – 2020 (%). DAMA discharge without medical advice, IVT intravenous thrombolytic therapy, MT mechanical thrombectomy

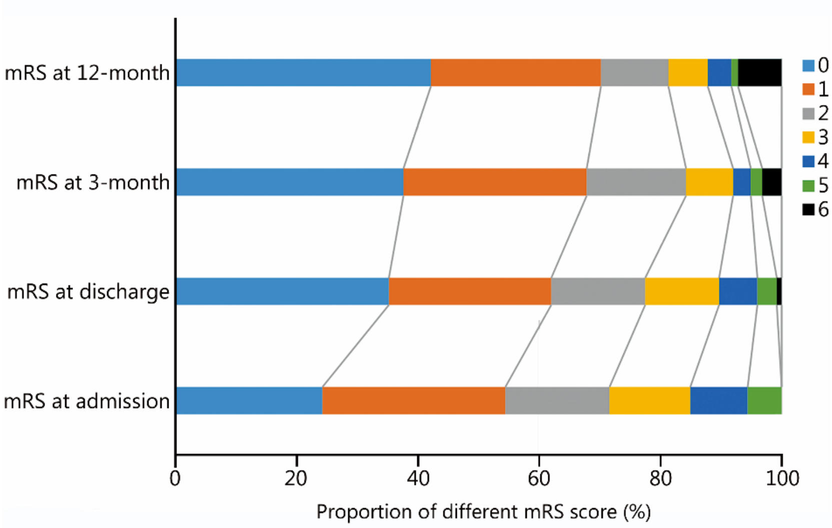

Fig. S10 mRS scores of ischemic stroke patients at different time points (%). mRS modified Rankin Scale

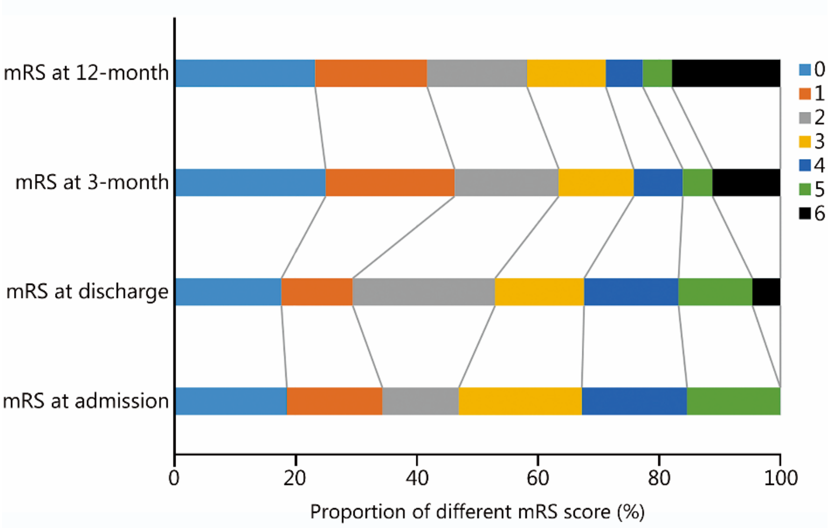

Fig. S11 mRS scores of intracerebral hemorrhage patients at different time points (%). mRS modified Rankin Scale

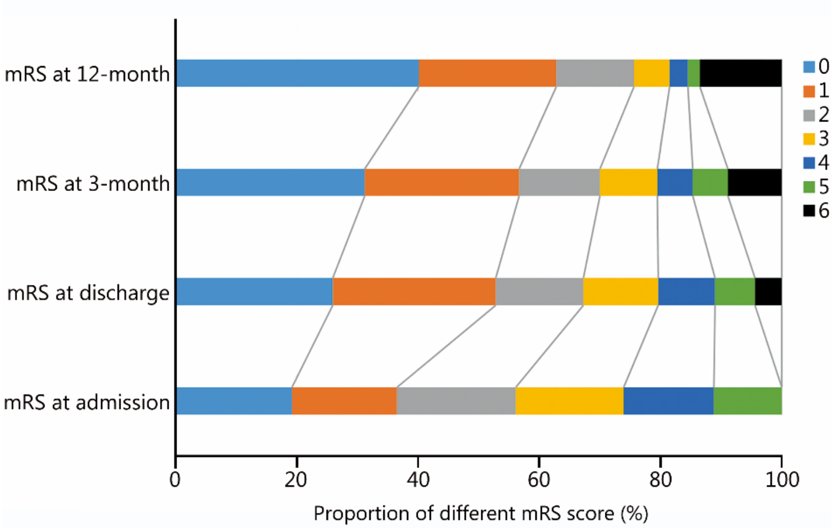

Fig. S12 mRS scores of subarachnoid hemorrhage patients at different time points (%). mRS modified Rankin Scale
