# Supplementary material for: China stroke surveillance report 2021
Source: Mil Med Res. 2023 Jul 19;10:33. doi: 10.1186/s40779-023-00463-x (PMC10355019; doi:10.1186/s40779-023-00463-x)
Supplement: Supplementary file 2 — Additional file 2: Table S1. Thrombolytic therapy for acute ischemic stroke in different provinces, 2019–2020. Table S2. Mechanical thrombectomy for acute ischemic stroke in different provinces, 2019–2020. Table S3. Bridging therapy for acute ischemic stroke in different provinces, 2019–2020 [file 40779_2023_463_MOESM2_ESM.pdf]

Table S1 Thrombolytic therapy for acute ischemic stroke in different provinces, 2019 – 2020

	IS patients	TP	IVTR	IHR	DAMA rate	Mortality rate
**China**	5,987,612	188,648	3.2 (3.1 – 3.2)	3.2 (3.1 – 3.3)	7.2 (7.1 – 7.3)	1.7 (1.7 – 1.8)
Anhui	250,808	6049	2.4 (2.4 – 2.4)	4.1 (3.6 – 4.6)	5.9 (5.3 – 6.5)	1.9 (1.9 – 2.2)
Beijing	43,469	1942	4.5 (4.3 – 4.5)	1.6 (1.1 – 2.2)	2.7 (2.0 – 3.5)	2.4 (2.4 – 3.0)
CC	24,042	-	-	-	-	-
Fujian	76,988	3325	4.3 (4.2 – 4.3)	5.2 (4.4 – 6.0)	9.9 (8.9 – 10.9)	1.3 (1.3 – 1.7)
Gansu	91,548	2062	2.3 (2.2 – 2.3)	3.4 (2.7 – 4.2)	4.9 (4.0 – 5.9)	1.9 (1.9 – 2.5)
Guangdong	327,269	8289	2.5 (2.5 – 2.5)	2.6 (2.5 – 2.7)	7.3 (6.7 – 7.8)	1.8 (1.5 – 2.1)
Guangxi	124,788	4027	3.2 (3.1 – 3.2)	3.5 (3.4 – 3.7)	5.2 (4.5 – 5.9)	2.4 (1.9 – 2.9)
Guizhou	64,210	2941	4.6 (4.4 – 4.6)	4.3 (3.5 – 5.0)	15.2 (13.9 – 16.5)	1.6 (1.6 – 2.1)
Hainan	86,195	1435	1.7 (1.6 – 1.7)	3.3 (2.4 – 4.3)	10.9 (9.3 – 12.6)	1.6 (1.6 – 2.3)
Hebei	502,950	15,205	3.0 (3.0 – 3.0)	2.1 (1.8 – 2.3)	8.3 (7.8 – 8.7)	0.8 (0.8 – 0.9)
Henan	774,938	21,639	2.8 (2.8 – 2.8)	2.1 (1.9 – 2.3)	4.1 (3.8 – 4.4)	1.1 (1.1 – 1.3)
Heilongjiang	339,450	6236	1.8 (1.8 – 1.8)	3.0 (2.6 – 3.4)	7.1 (6.4 – 7.7)	2.5 (2.5 – 2.9)
Hubei	234,095	8459	3.6 (3.5 – 3.6)	5.0 (4.5 – 5.4)	5.2 (4.8 – 5.7)	2.7 (2.7 – 3.1)
Hunan	121,324	3724	3.1 (3.0 – 3.1)	3.9 (3.3 – 4.6)	6.3 (5.5 – 7.0)	0.8 (0.8 – 1.1)
Jilin	143,214	3716	2.6 (2.5 – 2.6)	1.8 (1.4 – 2.3)	4.4 (3.7 – 5.0)	2.7 (2.7 – 3.2)
Jiangsu	316,558	8365	2.6 (2.6 – 2.6)	4.3 (3.8 – 4.7)	4.7 (4.3 – 5.2)	1.1 (1.1 – 1.3)
Jiangxi	92,841	3647	3.9 (3.8 – 3.9)	4.9 (4.2 – 5.6)	9.8 (8.8 – 10.7)	1.2 (1.2 – 1.5)
Liaoning	330,390	11,631	3.5 (3.5 – 3.5)	3.0 (2.7 – 3.4)	4.9 (4.5 – 5.3)	3.5 (3.5 – 3.9)
IM	161,894	4794	3.0 (2.9 – 3.0)	3.3 (2.8 – 3.8)	13.8 (12.8 – 14.8)	2.0 (2.0 – 2.4)
Ningxia	25,372	146	0.6 (0.5 – 0.6)	-	4.1 (0.9 – 7.3)	2.1 (2.1 – 4.4)
Qinghai	8216	41	0.5 (0.3 – 0.5)	7.3 (-0.7 to 15.3)	26.8 (13.3 – 40.4)	4.9 (4.9 – 11.5)
Shandong	804,918	25,302	3.1 (3.1 – 3.1)	2.3 (2.1 – 2.5)	7.5 (7.2 – 7.8)	1.5 (1.5 – 1.6)
Shanxi	158,115	6268	4.0 (3.9 – 4.0)	2.2 (1.8 – 2.5)	4.8 (4.3 – 5.3)	0.8 (0.8 – 1.0)
Shaanxi	118,611	2039	1.7 (1.6 – 1.7)	4.1 (3.3 – 5.0)	7.4 (6.3 – 8.5)	1.9 (1.9 – 2.5)
Shanghai	50,437	853	1.7 (1.6 – 1.7)	2.9 (1.8 – 4.1)	1.1 (0.4 – 1.7)	5.9 (5.4 – 6.4)
Sichuan	211,479	8136	3.8 (3.8 – 3.8)	4.8 (4.3 – 5.2)	12.9 (12.1 – 13.6)	2.1 (2.1 – 2.5)
Tianjin	98,936	8188	8.3 (8.1 – 8.3)	2.3 (2.0 – 2.7)	10.5 (9.8 – 11.2)	1.1 (1.1 – 1.4)
Tibet	373	-	-	-	-	-
Xinjiang	59,978	3057	5.1 (4.9 – 5.1)	3.6 (3.0 – 4.3)	5.2 (4.4 – 6.0)	3.8 (3.8 – 4.4)
Yunnan	122,378	6272	5.1 (5.0 – 5.1)	4.7 (4.2 – 5.3)	9.7 (9.0 – 10.4)	1.7 (1.7 – 2.0)
Zhejiang	177,197	8127	4.6 (4.5 – 4.6)	5.1 (4.7 – 5.6)	5.4 (4.9 – 5.9)	1.1 (1.1 – 1.3)
Chongqing	44,631	2733	6.1 (5.9 – 6.1)	4.6 (3.8 – 5.4)	14.1 (12.7 – 15.4)	2.6 (2.6 – 3.2)

The results were presented as number or percentage (95% CI). "-" indicates a lack of data from Xinjiang Production and Construction Corps and Tibet. *IS* ischemic stroke, *TP* thrombolytic patient, *CC* Xinjiang Production and Construction Corps, *IM* Inner Mongolia, *IHR* intracranial hemorrhage rate, *IVT*R intravenous thrombolysis rate, *DAMA* discharge against medical advice

Table S2 Mechanical thrombectomy for acute ischemic stroke in different provinces, 2019 – 2020

	IS patients	TP	MTR	IHR	DAMA rate	Mortality rate
**China**	5,987,612	49,845	0.8 (0.8 – 0.8)	7.7 (7.5 – 8.0)	11.5 (11.2 – 11.8)	5.0 (4.8 – 5.2)
Anhui	250,808	1733	0.7 (0.7 – 0.7)	8.4 (7.1 – 9.7)	11.7 (10.1 – 13.2)	3.5 (2.7 – 4.4)
Beijing	43,469	730	1.7 (1.7 – 1.7)	6.4 (4.7 – 8.2)	1.6 (0.7 – 2.6)	5.8 (4.1 – 7.4)
CC	24,042	-	-	-	-	-
Fujian	76,988	1827	2.4 (2.4 – 2.4)	7.7 (6.5 – 8.9)	8.8 (7.5 – 10.1)	2.0 (1.4 – 2.7)
Gansu	91,548	515	0.6 (0.6 – 0.6)	10.9 (8.2 – 13.6)	15.7 (12.6 – 18.9)	4.5 (2.7 – 6.3)
Guangdong	327,269	4703	1.4 (1.4 – 1.4)	8.2 (7.4 – 9.0)	9.8 (9.0 – 10.7)	4.4 (3.8 – 5.0)
Guangxi	124,788	1901	1.5 (1.5 – 1.5)	7.4 (6.2 – 8.6)	10.4 (9.0 – 11.8)	6.4 (5.3 – 7.5)
Guizhou	64,210	905	1.4 (1.4 – 1.4)	11.3 (9.2 – 13.3)	20.7 (18.0 – 23.3)	5.7 (4.2 – 7.3)
Hainan	86,195	716	0.8 (0.8 – 0.8)	8.2 (6.2 – 10.3)	11.0 (8.7 – 13.3)	3.1 (1.8 – 4.3)
Hebei	502,950	1921	0.4 (0.4 – 0.4)	5.9 (4.9 – 7.0)	17.6 (15.9 – 19.3)	3.1 (2.3 – 3.9)
Henan	774,938	5101	0.7 (0.7 – 0.7)	6.2 (5.6 – 6.9)	12.0 (11.1 – 12.9)	4.1 (3.6 – 4.6)
Heilongjiang	339,450	948	0.3 (0.3 – 0.3)	12.6 (10.4 – 14.7)	9.0 (7.1 – 10.8)	7.6 (5.9 – 9.3)
Hubei	234,095	1750	0.7 (0.7 – 0.7)	12.6 (11.0 – 14.1)	8.3 (7.0 – 9.6)	8.1 (6.8 – 9.4)
Hunan	121,324	1172	1.0 (1.0 – 1.0)	9.3 (7.6 – 11.0)	11.5 (9.7 – 13.3)	2.0 (1.2 – 2.8)
Jilin	143,214	1688	1.2 (1.2 – 1.2)	5.2 (4.1 – 6.2)	4.6 (3.6 – 5.6)	6.5 (5.3 – 7.7)
Jiangsu	316,558	3965	1.3 (1.3 – 1.3)	8.9 (8.0 – 9.8)	10.8 (9.8 – 11.7)	2.7 (2.2 – 3.3)
Jiangxi	92,841	1623	1.7 (1.7 – 1.7)	7.8 (6.5 – 9.1)	15.5 (13.7 – 17.2)	2.5 (1.7 – 3.2)
Liaoning	330,390	1900	0.6 (0.6 – 0.6)	8.4 (7.1 – 9.6)	7.4 (6.2 – 8.6)	15.3 (13.6 – 16.9)
IM	161,894	484	0.3 (0.3 – 0.3)	9.3 (6.7 – 11.9)	17.8 (14.4 – 21.2)	6.2 (4.1 – 8.3)
Ningxia	25,372	30	0.1 (0.1 – 0.1)	0	6.7 (-2.3 to 15.6)	0
Qinghai	8216	12	0.1 (0.1 – 0.1)	16.7 (-4.4 to 37.8)	33.3 (6.7 – 60.0)	25.0 (0.5 – 49.5)
Shandong	804,918	4693	0.6 (0.6 – 0.6)	5.9 (5.2 – 6.6)	12.9 (11.9 – 13.9)	5.9 (5.3 – 6.6)
Shanxi	158,115	710	0.4 (0.4 – 0.4)	5.1 (3.5 – 6.7)	9.4 (7.3 – 11.6)	4.6 (3.1 – 6.2)
Shaanxi	118,611	962	0.8 (0.8 – 0.8)	10.8 (8.8 – 12.8)	14.2 (12.0 – 16.4)	5.0 (3.6 – 6.4)
Shanghai	50,437	399	0.8 (0.8 – 0.8)	3.0 (1.3 – 4.7)	1.3 (0.2 – 2.3)	13.0 (9.7 – 16.3)
Sichuan	211,479	2793	1.3 (1.3 – 1.3)	8.3 (7.2 – 9.3)	17.4 (16.0 – 18.8)	5.2 (4.4 – 6.1)
Tianjin	98,936	745	0.8 (0.8 – 0.8)	5.1 (3.5 – 6.7)	9.7 (7.5 – 11.8)	4.0 (2.6 – 5.4)
Tibet	373	-	-	-	-	-
Xinjiang	59,978	1120	1.9 (1.9 – 1.9)	8.3 (6.7 – 9.9)	11.4 (9.6 – 13.3)	10.4 (8.7 – 12.2)
Yunnan	122,378	738	0.6 (0.6 – 0.6)	10.4 (8.2 – 12.6)	15.6 (13.0 – 18.2)	5.3 (3.7 – 6.9)
Zhejiang	177,197	3414	1.9 (1.9 – 1.9)	7.3 (6.4 – 8.1)	9.0 (8.0 – 9.9)	2.4 (1.9 – 2.9)
Chongqing	44,631	647	1.4 (1.4 – 1.4)	6.2 (4.3 – 8.0)	19.5 (16.4 – 22.5)	4.9 (3.3 – 6.6)

The results were presented as number or percentage (95% CI). "-" indicates a lack of data from Xinjiang Production and Construction Corps and Tibet. *IS* ischemic stroke, *TP* thrombectomy patient, *MTR* Mechanical thrombectomy rate, *IHR* intracranial hemorrhage rate, *DAMA* discharge against medical advice, *CC* Xinjiang Production and Construction Corps, *IM* Inner Mongolia

Table S3 Bridging therapy for acute ischemic stroke in different provinces, 2019-2020

	IS patients	BP	BTR	IHR	DAMA rate	Mortality rate
**China**	5,987,612	14,087	0.2 (0.2 – 0.2)	12.9 (12.3 – 13.4)	11.0 (10.5 – 11.5)	5.8 (5.4 – 6.2)
Anhui	250,808	522	0.2 (0.2 – 0.2)	13.4 (10.5 – 16.3)	11.5 (8.8 – 14.2)	5.6 (3.6 – 7.5)
Beijing	43,469	204	0.5 (0.5 – 0.5)	7.4 (3.8 – 10.9)	2.0 (0.1 – 3.9)	9.8 (5.7 – 13.9)
CC	24,042	0	-	-	-	-
Fujian	76,988	405	0.5 (0.5 – 0.5)	14.1 (10.7 – 17.5)	10.4 (7.4 – 13.3)	3.2 (1.5 – 4.9)
Gansu	91,548	199	0.2 (0.2 – 0.2)	13.6 (8.8 – 18.3)	12.1 (7.5 – 16.6)	3.5 (1.0 – 6.1)
Guangdong	327,269	1331	0.4 (0.4 – 0.4)	5.9 (4.6 – 7.1)	9.5 (7.9 – 11.0)	3.7 (2.7 – 4.7)
Guangxi	124,788	606	0.5 (0.5 – 0.5)	26.7 (23.2 – 30.3)	9.1 (6.8 – 11.4)	5.9 (4.1 – 7.8)
Guizhou	64,210	342	0.5 (0.5 – 0.5)	16.7 (12.7 – 20.6)	19.0 (14.8 – 23.2)	4.4 (2.2 – 6.6)
Hainan	86,195	202	0.2 (0.2 – 0.2)	13.4 (8.7 – 18.1)	12.9 (8.3 – 17.5)	5.9 (2.7 – 9.2)
Hebei	502,950	435	0.1 (0.1 – 0.1)	9.9 (7.1 – 12.7)	17.9 (14.3 – 21.5)	4.1 (2.3 – 6.0)
Henan	774,938	1455	0.2 (0.2 – 0.2)	7.5 (6.1 – 8.8)	10.0 (8.4 – 11.5)	4.1 (3.0 – 5.1)
Heilongjiang	339,450	243	0.1 (0.1 – 0.1)	20.6 (15.5 – 25.7)	9.5 (5.8 – 13.1)	7.4 (4.1 – 10.7)
Hubei	234,095	490	0.2 (0.2 – 0.2)	16.9 (13.6 – 20.3)	7.3 (5.0 – 9.7)	12.4 (9.5 – 15.4)
Hunan	121,324	365	0.3 (0.3 – 0.3)	14.8 (11.2 – 18.4)	11.0 (7.8 – 14.2)	3.0 (1.3 – 4.8)
Jilin	143,214	225	0.2 (0.2 – 0.2)	11.6 (7.4 – 15.7)	1.3 (-0.2 to 2.8)	12.4 (8.1 – 16.8)
Jiangsu	316,558	985	0.3 (0.3 – 0.3)	13.4 (11.3 – 15.5)	9.0 (7.2 – 10.8)	3.5 (2.3 – 4.6)
Jiangxi	92,841	425	0.5 (0.5 – 0.5)	15.1 (11.7 – 18.5)	16.7 (13.2 – 20.3)	2.1 (0.7 – 3.5)
Liaoning	330,390	592	0.2 (0.2 – 0.2)	15.9 (12.9 – 18.8)	6.4 (4.4 – 8.4)	16.6 (13.6 – 19.5)
IM	161,894	216	0.1 (0.1 – 0.1)	13.9 (9.3 – 18.5)	13.4 (8.9 – 18.0)	6.5 (3.2 – 9.8)
Ningxia	25,372	2	0	0	0	0
Qinghai	8216	1	0	0	100	0
Shandong	804,918	1651	0.2 (0.2 – 0.2)	7.8 (6.5 – 9.1)	11.6 (10.1 – 13.2)	5.7 (4.6 – 6.8)
Shanxi	158,115	177	0.1 (0.1 – 0.1)	6.8 (3.1 – 10.5)	9.6 (5.3 – 13.9)	7.9 (3.9 – 11.9)
Shaanxi	118,611	178	0.2 (0.2 – 0.2)	15.7 (10.4 – 21.1)	10.1 (5.7 – 14.5)	6.2 (2.6 – 9.7)
Shanghai	50,437	159	0.3 (0.3 – 0.3)	6.3 (2.5 – 10.1)	0.6 (-0.6 to 1.9)	9.4 (4.9 – 14.0)
Sichuan	211,479	798	0.4 (0.4 – 0.4)	15.8 (13.3 – 18.3)	18.3 (15.6 – 21.0)	7.0 (5.2 – 8.8)
Tianjin	98,936	194	0.2 (0.2 – 0.2)	11.3 (6.9 – 15.8)	14.4 (9.5 – 19.4)	4.6 (1.7 – 7.6)
Tibet	373	0	-	-	-	-
Xinjiang	59,978	324	0.5 (0.5 – 0.5)	14.5 (10.7 – 18.3)	10.5 (7.2 – 13.8)	11.4 (8.0 – 14.9)
Yunnan	122,378	207	0.2 (0.2 – 0.2)	12.6 (8.0 – 17.1)	14.5 (9.7 – 19.3)	5.8 (2.6 – 9.0)
Zhejiang	177,197	934	0.5 (0.5 – 0.5)	13.5 (11.3 – 15.7)	9.9 (7.9 – 11.8)	3.0 (1.9 – 4.1)
Chongqing	44,631	220	0.5 (0.5 – 0.5)	10.9 (6.8 – 15.0)	16.4 (11.5 – 21.3)	6.4 (3.1 – 9.6)

The results were presented as number or percentage (95% CI). "-" indicates a lack of data from Xinjiang Production and Construction Corps and Tibet. *IS* ischemic stroke, *BP* bridging patient, *BTR* Bridging therapy rate, *IHR* intracranial hemorrhage rate, *DAMA* discharge against medical advice, *CC* Xinjiang Production and Construction Corps, *IM* Inner Mongolia
